# High pathogenicity avian influenza A (H5N1) viruses isolated from poultry and wild birds in Japan during the 2024–2025 season

**DOI:** 10.1128/spectrum.00293-26

**Published:** 2026-07-14

**Authors:** Yoshihiro Takadate, Hayate Nishiura, Asuka Kumagai, Junki Mine, Ryota Tsunekuni, Saki Sakuma, Kosuke Soda, Mana Esaki, Kosuke Okuya, Kei Nabeshima, Takahiro Hiono, Hiroki Takakuwa, Tatsufumi Usui, Makoto Ozawa, Manabu Onuma, Norikazu Isoda, Yoshihiro Sakoda, Yuko Uchida, Kohtaro Miyazawa

**Affiliations:** 1 Emerging Virus Group, Division of Zoonosis Research, National Institute of Animal Health, National Agriculture and Food Research Organization 13516, Tsukuba, Ibaraki, Japan; 2 Avian Infectious Disease and Global Health Research Center, Tottori University 13114, Tottori, Japan; 3 Joint Graduate School of Veterinary Science, Kagoshima University 12851, Kagoshima, Japan; 4 Joint Faculty of Veterinary Medicine, Kagoshima University 12851, Kagoshima, Japan; 5 Biodiversity Division, National Institute for Environmental Studies 13585, Tsukuba, Japan; 6 Laboratory of Microbiology, Department of Disease Control, Faculty of Veterinary Medicine, Hokkaido University 12810, Sapporo, Japan; 7 One Health Research Center, Hokkaido University 12810, Sapporo, Japan; 8 International Collaboration Unit, International Institute for Zoonosis Control, Hokkaido University 12810, Sapporo, Hokkaido, Japan; 9 Hokkaido University Institute for Vaccine Research and Development (HU-IVReD), Hokkaido University 12810, Sapporo, Hokkaido, Japan; 10 Faculty of Life Sciences, Kyoto Sangyo University 26336, Kyoto, Japan; National Chung Hsing University, Taiwan, Taiwan

**Keywords:** high pathogenicity avian influenza virus, 2024-2025 season, poultry, wild birds, H5N1, Japan, phylogenetic analysis, pathogenic analysis, bovine mammary epithelial cells

## Abstract

H5N1 high pathogenicity avian influenza (HPAI) viruses of clade 2.3.4.4b continue to spread globally among wild birds and poultry, and diversify through gene reassortment. Since 2024, spillovers of H5N1 viruses have been reported in dairy cattle in the United States. Given the numerous HPAI outbreaks in poultry and wild birds in Japan during the 2024–2025 season, we aimed to examine the genetic diversity, pathogenicity, and host range of these viruses. Phylogenetic analyses revealed that all viruses in the 2024–2025 season belonged to G2d and G2c subgroups in clade 2.3.4.4b and were divided into 11 genotypes (G2d: six genotypes, G2c: five genotypes). Of these, G2d-0 was the dominant genotype in both poultry and wild birds. Novel genotypes emerged through reassortment between G2d and G2c viruses. Experimental infection of chickens revealed comparable pathogenicity among genotypes, although the amounts of viral shedding were high in chickens inoculated with G2d-0 and G2c-13 viruses, which were genotypes confirmed in multiple outbreaks in the 2024–2025 season, suggesting high transmission potential. *In vitro* assays revealed markedly lower infectivity of Japanese isolates in bovine mammary epithelial cells compared with that of the B3.13 genotype responsible for dairy cattle outbreaks in the United States, indicating limited risk of cattle infection. Together, these findings underscore ongoing genetic diversification of HPAI viruses in G2d and G2c subgroups and highlight the importance of continued *in vivo* and *in vitro* characterization of these viruses to monitor changes in genotypes, pathogenicity, and potential risk of spillover to cattle.

**IMPORTANCE:**

H5N1 high pathogenicity avian influenza (HPAI) viruses have spread in poultry and wild birds worldwide. H5N1 viruses have also spread in dairy cattle in the United States since 2024. Japan reported numerous HPAI cases during the 2024–2025 season. Herein, phylogenetic analyses using strains isolated from poultry and wild birds enabled a detailed investigation of their genetic backgrounds, which is essential for elucidating introduction routes and evolution. Investigation of pathogenicity may help predict the outbreak frequency. This study revealed that HPAI viruses of multiple genotypes had been introduced into Japan, and chickens infected with genotypes detected from continuous outbreaks exhibited high virus shedding. In addition, comparing infectivity in bovine cells revealed the risk of cattle infection. H5N1 infection had not been reported in cattle in Japan, and our results suggest that the risk remains low. These integrated analyses provide key insights for developing effective strategies against HPAI outbreaks.

## INTRODUCTION

H5 high pathogenicity avian influenza (HPAI) viruses of the A/goose/Guangdong/1/1996 (H5N1) (Gs/Gd) lineage significantly affect wild birds and poultry and are divided into 10 clades (0–9) and multiple subclades based on hemagglutinin (HA) genes (1, 2). H5Nx HPAI viruses of clade 2.3.4.4b, which may have originated from a virus initially identified in the Netherlands in autumn 2020, are currently dominant in wild birds and poultry worldwide, excluding Oceania (3, 4). Japan has also experienced a significant number of outbreaks in poultry farms caused by HPAI viruses belonging to a G2 group in clade 2.3.4.4b since the 2020–2021 season (5–9). Phylogenetic analyses of the HA gene revealed that the G2 group comprises viruses that are genetically similar to those that circulated in Europe during late 2020 (10). The G2 group in Japan is further divided into four subgroups: G2a, G2b, G2c, and G2d. In contrast, at least six distinct viruses classified within clade 2.3.4.4b from Europe and Asia (genotypes A1–A6) have been introduced in the United States since late 2021 (11–13). After the introduction of Eurasian HPAI viruses into North America, genotypes B3.13 and D1.1 emerged due to the reassortment events of A1 and A3 viruses with low pathogenicity avian influenza (LPAI) viruses circulating in migratory birds in North America, respectively (14–16). Genotype D1.1 is responsible for the ongoing outbreaks in poultry and wild birds in the United States. Both genotypes, D1.1 and B3.13, have been reported to transmit to dairy cattle and humans in the United States (14). The G2d subgroup in Japan shares the HA gene with genotypes A3 and D1.1 in the United States. In particular, genotype A3 corresponds to genotype G2d-0 in Japan (13, 17).

Many genotypes have been identified in the G2 group (6–8). Viruses belonging to the G2b and G2c subgroups possess internal genes derived from LPAI viruses in Asia and Russia (7, 8), whereas those belonging to the G2d subgroup possess internal genes derived from LPAI viruses in Asia, Russia, and North America (7). Phylogenetic analyses of individual genome segments have shown that genotype G2a-2 in Japan is identical to genotype A6 in the United States, clustering with H5N5 viruses isolated in Norway, Iceland, and other European regions, with no evidence of reassortment (18).

During the 2024–2025 season in Japan, 51 outbreaks were identified in poultry farms in 14 prefectures, and 227 cases across 19 prefectures were reported among wild birds and environmental samples (19). In this study, we present phylogenetic analyses of all eight segments to clarify the genetic characteristics of the H5 HPAI viruses that invaded Japan during this season. Additionally, we investigated the virological properties of five representative virus strains isolated from poultry farms by experimental infection of chickens, because multiple poultry outbreaks and wild bird cases with diverse genetic features may result in differences in pathogenicity even within clade 2.3.4.4b (6, 8, 20–22). We also compared the susceptibility of bovine mammary epithelial cells (BMECs) to two representative G2c and G2d virus strains.

## MATERIALS AND METHODS

### Virus isolation and whole-genome sequencing of poultry samples

Tracheal or cloacal swabs were collected from poultry premises where outbreaks occurred and inoculated into embryonated chicken eggs at the diagnostic laboratories of the respective prefectural Animal Health Service centers. After 48 h of incubation at 37°C, allantoic fluids exhibiting HA activity were transported to the National Institute of Animal Health (NIAH) for further analysis.

Whole-genome sequencing of the isolates was performed using the Illumina MiSeq platform. cDNA libraries were prepared using a NEBNext Ultra II RNA Library Prep Kit for Illumina (New England Biolabs; https://www.neb.com/en-us/?country=en-us), and sequencing was conducted using a MiSeq Reagent Nano Kit v2 (Illumina; https://www.illumina.com/). Raw sequencing reads were assembled into consensus sequences using CLC Genomics Workbench v.23.0.2 (Qiagen; https://digitalinsights.qiagen.com/) and FluGAS v.2.5 (World Fusion; https://www.w-fusion.com/). All newly obtained sequences were deposited in the Global Initiative on Sharing All Influenza Data (GISAID) database (http://platform.gisaid.org; accessed 10 November 2025). Isolate IDs are listed in Table S1.

### Virus isolation and whole-genome sequencing of wild bird samples

Tracheal or cloacal swabs were obtained from carcasses of wild birds confirmed to be HPAI-positive via HA gene sequence detection conducted by the National Institute for Environmental Studies, Japan. These samples were transported to designated laboratories at NIAH, Hokkaido University, Tottori University, and Kagoshima University. Virus isolation and whole-genome sequencing were performed following the standard protocols of each institution. At Hokkaido University and Kagoshima University, virus isolation and sequencing were performed following previously described protocols (23, 24). At NIAH, virus isolation from wild bird samples and sequencing were conducted using protocols identical to those used for poultry samples. At Tottori University, virus isolation was conducted following a previously described protocol (25), and viral RNA was extracted using the RNeasy Mini Kit (QIAGEN; https://www.qiagen.com/us). The viral RNA was subsequently sent to NIAH for whole-genome sequencing. All viral sequences analyzed in this study were submitted to the GISAID database (Table S2).

### Phylogenetic analyses

Nucleotide sequences of avian influenza viruses (AIVs) were downloaded from GISAID (deposited prior to 12 August 2025). In total, 97 poultry-derived and 176 wild bird-derived isolates sequenced in this study were aligned using MAFFT (26) and manually curated using BioEdit v7.2.5 (27). The final data set comprised 52,820 polymerase basic protein 2 (PB2), 51,249 polymerase basic protein 1 (PB1), 53,812 polymerase acidic protein (PA), 32,791 hemagglutinin (H5), 57,260 nucleoprotein (NP), 27,747 neuraminidase (N1), 58,099 matrix protein (MP), and 51,505 nonstructural protein (NS) gene sequences. Maximum likelihood phylogenetic trees were constructed using IQ-TREE 2 (28). The best-fit substitution models were automatically selected using ModelFinder (29) integrated within IQ-TREE. Branch support was evaluated using ultrafast bootstrap approximation (UFBoot) with 1,000 replicates (30), and the Shimodaira–Hasegawa approximate likelihood ratio test (SH-aLRT) (31) was performed to assess the robustness of the inferred tree topologies. Genotypic clusters were defined by SH-aLRT ≥80, UFBoot ≥95, and ≥98% nucleotide sequence identity within nodes. Exceptions (<98% identity) were permitted for a limited number of viruses if supported by epidemiological information.

### Animal experiments

Animal experiments were approved by the institutional ethics committee (approval no. R4-I014-NIAH-8) and conducted in biosafety level 3 facilities at the NIAH. Intranasal inoculation tests were performed as described previously (8). Four-week-old white leghorn chickens (L-M-6 strain) were purchased from Nisseiken (https://www.jp-nisseiken.co.jp/en/). Serum collected before inoculation was tested using an enzyme-linked immunosorbent assay (ELISA) (IDEXX Influenza A Ab Test kit; IDEXX Laboratories; https://www.idexx.com/en/) to confirm seronegativity of all chickens for the influenza A virus. HPAI strains representing each genotype during the 2024–2025 season were used: A/chicken/Chiba/24A2T/2024 (H5N1) (Chiba/24A2T, genotype G2d-0), A/chicken/Niigata/24B3T/2024 (H5N1) (Niigata/24B3T, G2d-5), A/chicken/Gifu/24A5T/2024 (H5N1) (Gifu/24A5T, G2d-6), A/chicken/Chiba/24B7T/2024 (H5N1) (Chiba/24B7T, G2c-13), and A/chicken/Ehime/24A3T/2024 (H5N1) (Ehime/24A3T, G2c-14). Similarly, HPAI strains representing each genotype in the 2022–2023 season were used: A/chicken/Kagawa/22B2T/2022 (H5N1) (G2c-6), A/chicken/Aichi/22A3T/2022 (H5N1) (Aichi/22A3T, G2c-8), and A/chicken/Hiroshima/22A6T/2022 (H5N1) (G2c-9). To calculate the 50% chicken lethal dose (CLD_50_) for each virus, chickens in cages were allocated to each group (five birds per cage) and provided with *ad libitum* access to food and water. The mean death time (MDT) was calculated as the mean survival time of chickens inoculated with 10^6^ 50% egg infectious dose (EID_50_) of each virus. Birds were monitored for 14 days following viral inoculation. Tracheal and cloacal swabs were collected at 1, 2, 3, 5, 7, 10, and 14 days post-inoculation (dpi) or upon death, suspended in 2 mL of Minimum Essential Medium (MEM) (Thermo Fisher Scientific; https://www.thermofisher.com/) (containing 0.5% bovine serum albumin, 25 mg/mL amphotericin B, 1,000 units/mL penicillin, 1,000 mg/mL streptomycin, 0.01 M HEPES, and 8.8 mg/mL NaHCO_3_), and stored at −80 °C. Swab samples were inoculated into 9- to 11-day-old embryonated eggs, and viral titers were determined as the EID_50_ using the Reed and Muench method (32). The detection limit was 0.2 log₁₀ EID_50_/mL. The area under the curve (AUC) of viral titers at each sampling point was calculated as described previously (33, 34). At the end of the experiment, serum was collected from surviving chickens and tested for infection. The presence of antibodies against the influenza A virus in the serum was examined using the method described above.

### Cells

Primary chicken embryo fibroblasts (CEFs) were prepared from 10-day-old embryonated chicken eggs as described previously with minor modifications (35). Primary CEFs and MDCK cells were cultured in Eagle’s MEM (Thermo Fisher Scientific) containing 10% fetal calf serum, penicillin (100 U/mL), streptomycin (100 μg/mL) (Thermo Fisher Scientific), and amphotericin B (25 μg/mL) (Thermo Fisher Scientific). Bovine mammary epithelial cells (BMECs) (36) were cultured in Dulbecco’s modified Eagle’s medium (DMEM) (Thermo Fisher Scientific) containing 10% fetal calf serum, penicillin (100 U/mL), streptomycin (100 μg/mL) (Thermo Fisher Scientific), GlutaMAX Supplement (2 mM) (Gibco; https://www.thermofisher.com/jp/ja/home/brands/gibco.htm.html), and ITS-G Supplement (1X) (FUJIFILM Wako Pure Chemical Corporation; https://www.fujifilm.com/ffwk/en). HEK293T cells were cultured in DMEM containing 10% fetal calf serum, penicillin (100 U/mL), and streptomycin (100 μg/mL) (Thermo Fisher Scientific).

### Generation of a H5N1 HPAI virus of genotype B3.13

Eight segmented genes of A/dairy cow/Texas/063224-24-1/2024 (H5N1) (Texas24), an H5N1 HPAI virus isolated from cattle milk in Texas, USA (37), were synthesized in pUC57-mini and pCC1-4k vectors (GenScript; https://www.genscript.com/) based on EPI_ISL_19155861 in GISAID. pUC57-mini was used for PB2, PB1, PA, HA, NA, NP, and NS, whereas pCC1-4k was used for MP. Genes were amplified with KOD One (TOYOBO; https://www.toyobo-global.com/) and cloned into pHW2000 (38) using an In-Fusion cloning kit (Takara; https://www.takarabio.com/). Plasmid sequences were confirmed by MiSeq (Illumina).

HEK293T and MDCK cells were co-cultured in 6-well plates and transfected with pHW2000 plasmids using TransIT-LT1 (Mirus Bio; https://www.mirusbio.com/). After 48 h, the supernatants were collected and inoculated into 9- to 11-day-old embryonated eggs, followed by incubation at 37 °C for 48 h. Rescued viruses were propagated in eggs for 24 h and stored at −80 °C. Viral genomes were sequenced using MiSeq to confirm the absence of unexpected mutations.

### Virus infection *in vitro*

CEFs and BMECs were seeded in 96-well plates at a concentration of 2.0 × 10^4^ cells/well. After 24 h, the cells were washed with infection medium (MEM containing 0.4% bovine serum albumin, 1% penicillin–streptomycin, 1% amphotericin B, and 3% MEM vitamin solution) and then inoculated with diluted viruses. At 1 h post-inoculation, the supernatants were removed, and the cells were washed with phosphate-buffered saline and cultured in infection medium. At 5 dpi, the viral titer was determined as the 50% of the tissue culture infectious dose (TCID_50_)/mL.

### Statistical analysis

Differences in MDT, viral titers in collected samples, and AUCs among strains were analyzed using the Kruskal–Wallis and Steel–Dwass tests. Differences in viral titers between tracheal and cloacal swabs were analyzed using the Wilcoxon signed–rank test. Differences in viral titers in avian and bovine cells were analyzed using one-way analysis of variance, followed by Tukey’s test. Statistical analyses were performed using the EZR software (Jichi Medical University, Japan) (39). Statistical significance was set at *P* < 0.05.

## RESULTS

### Phylogenetic analyses

Phylogenetic analysis of the HA gene revealed that all HPAI viruses isolated during the 2024–2025 season in Japan belonged to either the G2d or G2c subgroups within clade 2.3.4.4b.

**Fig 1:**
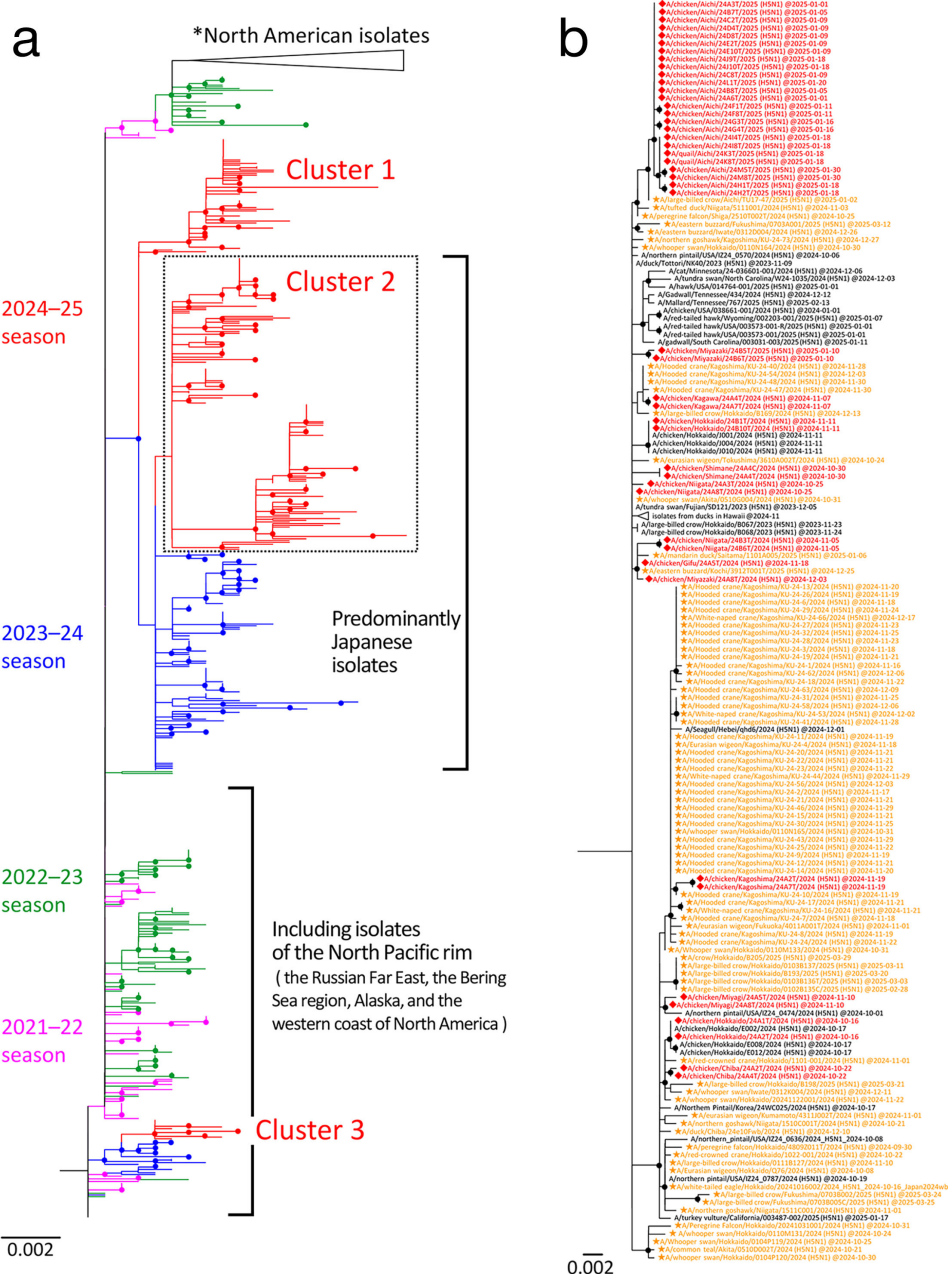
Phylogenetic tree of hemagglutinin (HA) gene sequences from the G2d subgroup. (**a**) Overall view. Colors indicate the season: magenta, 2021–2022; green, 2022–2023; blue, 2023–2024; and red, 2024–2025. (**b**) Magnified view of the cluster outlined by the dashed box in panel **a**. Red diamonds (◆) indicate isolates derived from Japanese poultry and analyzed at the National Institute of Animal Health (NIAH) during the 2024–2025 season, and yellow stars (✶) indicate isolates derived from wild birds collected in Japan during the same season. Filled circles (●) at nodes indicate Shimodaira–Hasegawa approximate likelihood ratio test (SH-aLRT)/bootstrap support values of ≥80/95.

Within the G2d subgroup, isolates from the 2024–2025 season formed three distinct clusters (Fig. 1a). Cluster 2 represented the major population, comprising poultry isolates (46 isolates derived from 25 cases) together with 84 wild-bird isolates collected during the same season (Fig. 1b), and clustered with isolates from the 2023–2024 season. Approximately 50% of the detections in wild birds were in cranes, and the remaining isolates were identified in multiple avian species, including crows and several raptors. This cluster was clearly distinct from the virus populations concurrently reported in North America. Although still limited in number, viruses belonging to Cluster 2 have also been reported from South Korea and China since December 2023, forming a monophyletic cluster with Japanese isolates. Clusters 1 and 3 consisted entirely of wild-bird isolates (75 isolates), which were mainly sampled in Hokkaido and other northern regions of Japan. Cluster 1 was characterized by isolates derived from crows in Hokkaido, as well as isolates from seabirds and marine mammals, such as seals and sea otters. Cluster 3 was closely related to isolates detected earlier, i.e., during the 2021–2022 and 2022–2023 seasons, indicating recurrent reintroduction of this group.

**Fig 2:**
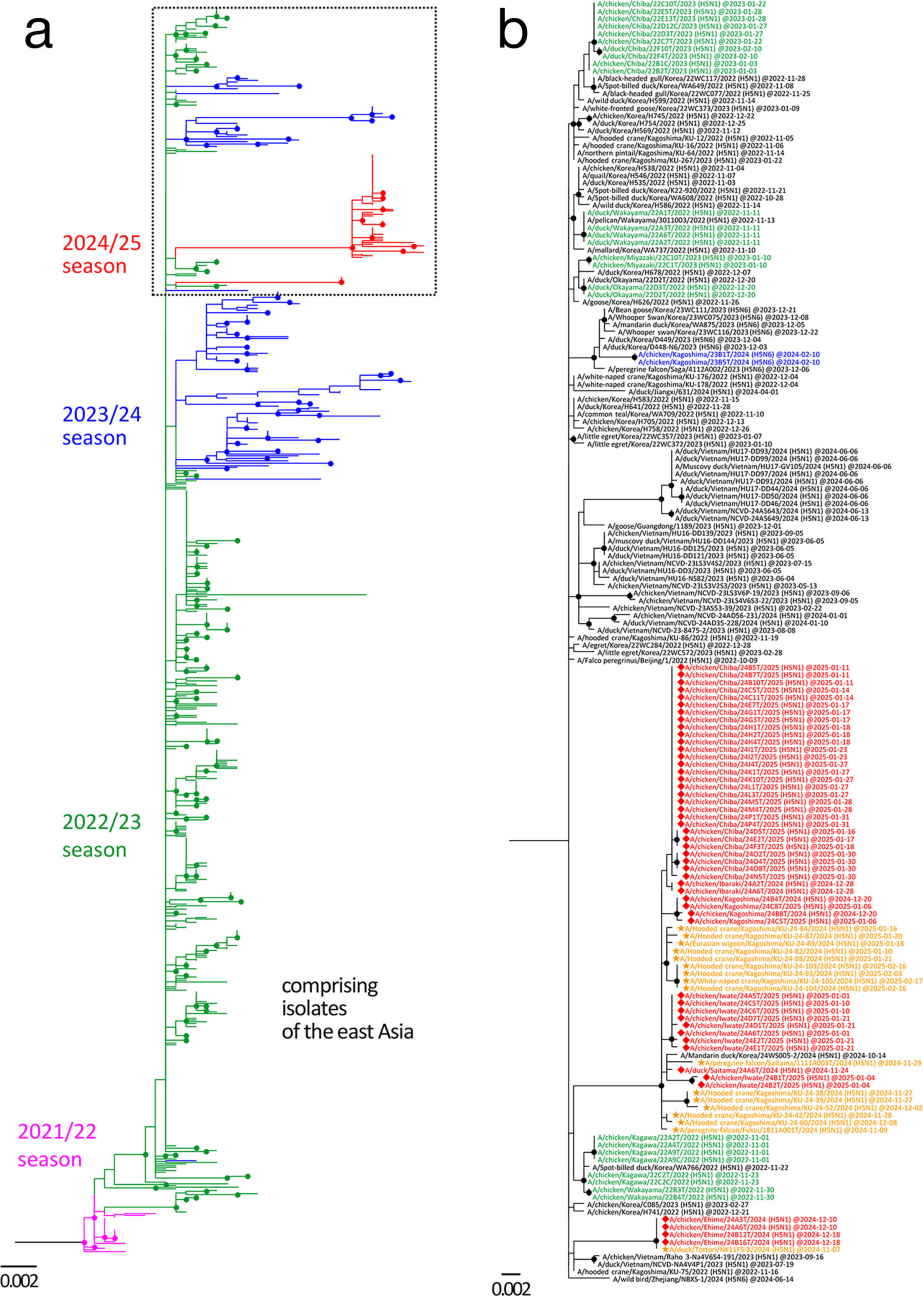
Phylogenetic tree of the hemagglutinin (HA) gene sequences from the G2c subgroup. (**a**) Overall view. Colors indicate the season: magenta, 2021–2022; green, 2022–2023; blue, 2023–2024; and red, 2024–2025. (**b**) Magnified view of the cluster outlined by the dashed box in panel **a**. Green, blue, and red represent isolates derived from Japanese poultry during the 2022–2023, 2023–2024, and 2024–2025 seasons, respectively. Diamonds (◆) indicate isolates analyzed at the National Institute of Animal Health (NIAH) during the 2024–2025 season, and yellow stars (✶) indicate isolates derived from wild birds collected in Japan during the same season. Filled circles (●) at nodes indicate Shimodaira–Hasegawa approximate likelihood ratio test (SH-aLRT)/bootstrap support values of ≥80/95.

Within the G2c subgroup, poultry isolates collected during the 2024–2025 season (51 isolates derived from 26 cases) formed a monophyletic cluster with isolates detected during the 2022–2023 season and exhibited greater genetic distance from those sampled during the 2023–2024 season (Fig. 2a). Wild-bird isolates belonging to the G2c subgroup (17 isolates) that were detected during the same season clustered with the poultry isolates (Fig. 2b). Most of these isolates were detected in hooded cranes, with a few cases identified in peregrine falcons and a Eurasian wigeon. The phylogenetic tree of the G2c subgroup consisted almost entirely of isolates detected in Asian countries, including Bangladesh, Vietnam, China, South Korea, and Japan.

Geospatial analysis of outbreak locations revealed that G2c-related poultry cases were broadly distributed across Japan, with a marked concentration in Chiba (16 cases) (Fig. S2). G2d-related cases also occurred nationwide, with a notable predominance in Aichi (13 cases). Wild-bird isolates were most frequently detected in Hokkaido and other northern regions.

Whole-genome analysis revealed that the G2c subgroup was classified into five genotypes (G2c-13–G2c-17) during the 2024–2025 season, whereas the G2d subgroup was divided into six genotypes (G2d-0 and G2d-5–G2d-9) (Fig. S1; Tables S1 and S2). Genotype names were assigned based on HA subgroups according to the scheme used in a previous study (7). G2d-0 was the predominant genotype in both poultry (22 out of 51 cases) and wild birds (155 out of 176 cases) during the 2024–2025 season. This genotype was detected in Japan for four consecutive seasons since the 2021–2022 season and had caused recurrent poultry outbreaks. Multiple outbreaks in Chiba and Aichi prefectures were confirmed to be caused by G2c-13 and G2d-0, respectively. In contrast, the number of poultry outbreaks and wild-bird cases due to other genotypes were limited (Table 1). Notably, two new genotypes resulting from the reassortment between the G2c and G2d subgroups were detected. G2d-6 harbored an HA gene from the G2d subgroup, whereas all other segments were identical to those of G2c-13. Similarly, G2d-8 contained seven gene segments derived from G2d, whereas its PA segment was identical to those of G2c-13, G2c-15, G2c-16, and G2c-17.

**TABLE 1:** Genotypes and genome-segment classifications of high pathogenicity avian influenza (HPAI) viruses isolated in Japan during the 2024-2025 season^*c*^

Subgroup	Genotype	Case number		Gene segment^*a*^							
		Poultry	Wild birds	PB2	PB1	PA	HA	NP	NA	MP	NS
G2d	G2d-0	22	155	G2d	G2d	G2d	G2d	G2d	G2d	G2d	G2d
	G2d-5	1	0	AIV-PB2-VII	G2d	AIV-PA-IX	G2d	AIV-NP-VII	G2d	G2d	AIV-NS-V
	G2d-6	2	0	AIV-PB2-VIII	AIV-PB1-VI	AIV-PA-X	G2d	AIV-NP-VIII	G2c	G2c	AIV-NS-IV
	G2d-7	0	1	AIV-PB2-I	G2d	AIV-PA-XI	G2d	G2d	G2d	G2d	G2d
	G2d-8	0	1	G2d	G2d	AIV-PA-X	G2d	G2d	G2d	G2d	G2d
	G2d-9	0	2	AIV-PB2-IX	G2d	AIV-PA-XII	G2d	AIV-NP-XI	G2d	G2d	G2d
G2c	G2c-12^*b*^	2	0	AIV-PB2-IV	AIV-PB1-II	AIV-PA-IV	G2c	AIV-NP-VI	N6	G2c	AIV-NS-III
	G2c-13	24	3	AIV-PB2-VIII	AIV-PB1-VI	AIV-PA-X	G2c	AIV-NP-VIII	G2c	G2c	AIV-NS-IV
	G2c-14	2	1	AIV-PB2-V	AIV-PB1-VII	AIV-PA-II	G2c	AIV-NP-III	G2c	G2c	AIV-NS-I
	G2c-15	0	3	AIV-PB2-VIII	AIV-PB1-VI	AIV-PA-X	G2c	AIV-NP-IX	G2c	G2c	AIV-NS-IV
	G2c-16	0	1	AIV-PB2-VIII	AIV-PB1-VI	AIV-PA-X	G2c	AIV-NP-X	G2c	G2c	AIV-NS-IV
	G2c-17	0	9	AIV-PB2-VIII	AIV-PB1-VIII	AIV-PA-X	G2c	AIV-NP-VIII	G2c	G2c	AIV-NS-IV

^*a*^ Genome segments were numbered according to the scheme used in a previous study (7).

^*b*^ G2c-12, a genotype identified from isolates collected during the 2023–2024 season (8), was not assigned each segment number in the previous study. In the present study, each genome segment was newly classified.

^*c*^ PB2, polymerase basic protein 2; PB1, polymerase basic protein 1; PA, polymerase acidic protein; HA, hemagglutinin; NP, nucleoprotein; NA, neuraminidase; MP, matrix protein; NS, nonstructural protein.

### Animal experiments

**Fig 3:**
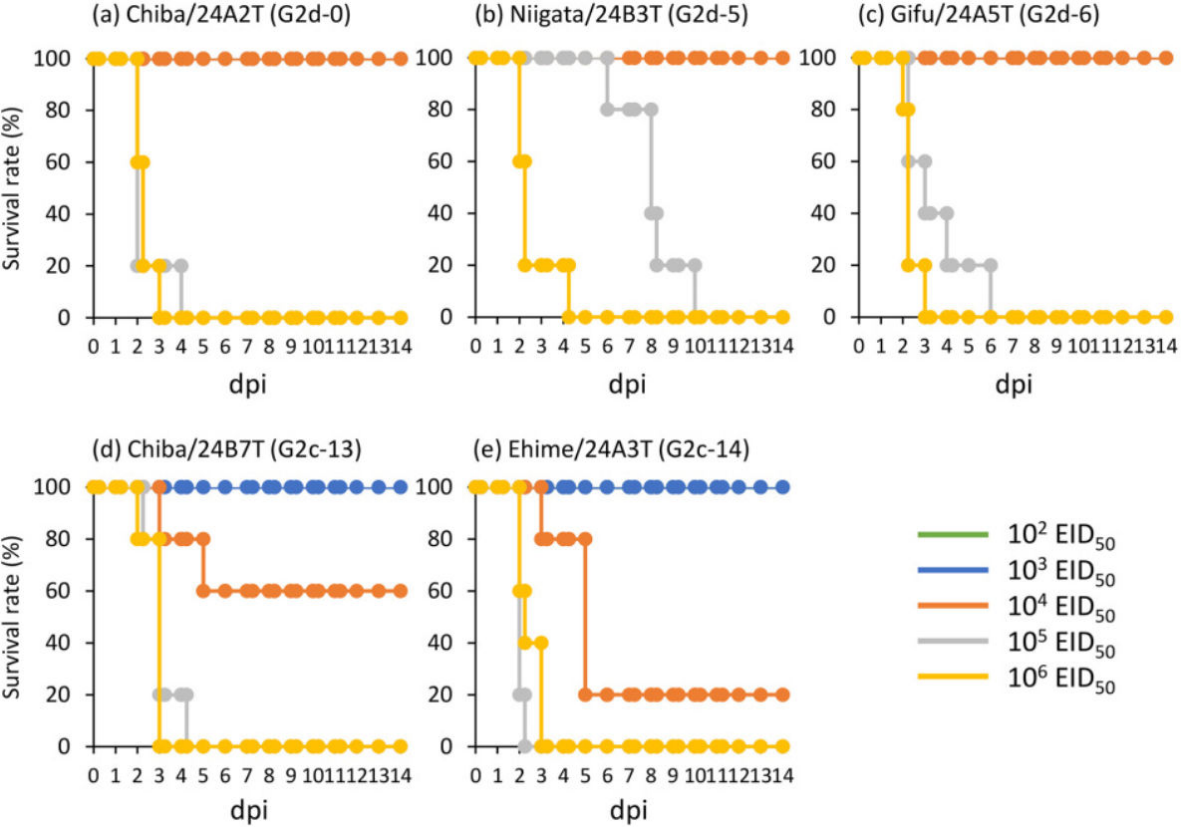
Survival rates of chickens inoculated with high pathogenicity avian influenza (HPAI) strains isolated during the 2024–2025 season. Survival rates of chickens inoculated with 10^2^, 10^3^, 10^4^, 10^5^, and 10^6^ 50% egg infectious dose (EID_50_) of representative virus strains: (**a**) Chiba/24A2T, (**b**) Niigata/24B3T, (**c**) Gifu/24A5T, (**d**) Chiba/24B7T, and (**e**) Ehime/24A3T are shown in green, blue, orange, gray, and yellow, respectively. dpi, days post-inoculation.

Chickens inoculated with 10^6^ EID_50_ of each HPAI virus during the 2024–2025 season died within 4 dpi (Fig. 3). At 10⁵ EID_50_, Chiba/24A2T, Gifu/24A5T, Chiba/24B7T, and Ehime/24A3T caused death within 6 dpi, and Niigata/24B3T caused death between 6 and 10 dpi (Fig. 3b). All chickens inoculated with 10⁴ EID_50_ of G2d viruses survived for 14 days, and two of five birds inoculated with Chiba/24B7T (G2c-13), and four of five birds inoculated with Ehime/24A3T (G2c-14) died within 5 dpi. Chickens inoculated with 10³ or 10² EID_50_ of G2d and G2c viruses survived and were seronegative for influenza A virus, as determined by ELISA. The CLD_50_ were 10^4.50^ EID_50_ (Chiba/24A2T, Niigata/24B3T, and Gifu/24A5T), 10^4.17^ EID_50_ (Chiba/24B7T), and 10^3.63^ EID_50_ (Ehime/24A3T) (Table 2). Although the lethal dose of Ehime/24A3T in chickens tended to be lower than that of the other strains, the CLD_50_ values of all the tested strains were within a 10-fold range.

**TABLE 2:** Virological characteristics of H5N1 high pathogenicity avian influenza (HPAI) viruses of G2d-0, G2d-5, G2d-6, G2c-13, and G2c-14 genotypes during the 2024–2025 season

Isolate	Genotype	Subtype	CLD_50_^*a*^ (log_10_EID_50_)	10^6^ EID_50_ inoculation group						
				MDT^*b*^		Mean maximum viral titer (log_10_EID_50_/mL)^*e*^		AUC of entire observation period^*f*^		
				Hpi^*c*^	Dpi^*d*^	Trachea	Cloaca	Trachea	Cloaca	Trachea and cloaca
Chiba/24A2T	G2d-0	H5N1	4.50	55.20	2.30	6.03	5.01	1,970,729	105,145	2,075,874
				(48.00–72.00)	(2.00–3.00)	(4.87–7.02)	(4.32–5.87)	(37,427–5,201,575)	(12,649–367,066)	(97,049–5,214,223)
Niigata/24B3T	G2d-5	H5N1	4.50	61.20	2.55	4.73	3.51	522,972	7,047	530,019
				(48.00–102.00)	(2.00–4.25)	(2.32–6.53)	(2.02–4.70)	(106–1,703,229)	(138–24,999)	(245–1,704,634)
Gifu/24A5T	G2d-6	H5N1	4.50	56.40	2.35	6.10	4.98	2,090,434	83,056	2,173,491
				(48.00–72.00)	(2.00–3.00)	(5.20–7.20)	(4.20–5.32)	(79,056–7,905,810)	(7,917–159,815)	(184,479–7,913,727)
Chiba/24B7T	G2c-13	H5N1	4.17	67.20	2.80	6.80	4.59	5,025,084	41,385	5,066,470
				(48.00–72.00)	(2.00–3.00)	(6.38–7.02)	(3.87–5.20)	(1,203,996–7,931,846)	(3,825–100,139)	(1,207,821–7,959,279)
Ehime/24A3T	G2c-14	H5N1	3.63	58.80	2.45	6.37	4.87	2,760,346	161,279	2,921,625
				(48.00–72.00)	(2.00–3.00)	(5.70–7.07)	(4.20–6.07)	(250,001–5,928,434)	(10,542–592,843)	(842,844–5,938,976)

^*a*^ CLD_50_, 50% chicken lethal dose; EID_50_, 50% egg infectious dose.

^*b*^ MDT, mean death time.

^*c*^ hpi, hours post-inoculation; values in parentheses, range of hpi.

^*d*^ dpi, days post-inoculation; values in parentheses, range of dpi.

^*e*^ Numbers in parentheses, range of maximum viral titers.

^*f*^ AUC, area under the curve; values in parentheses, range of AUC.

No apparent clinical sign was observed except depression and facial edema at high doses (Fig. S3). The MDT at 10⁶ EID_50_ ranged from 2.3 to 2.8 days (55.2–67.2 h) (Table 2). Although the MDT was not statistically different, Chiba/24B7T tended to show a longer MDT.

**Fig 4:**
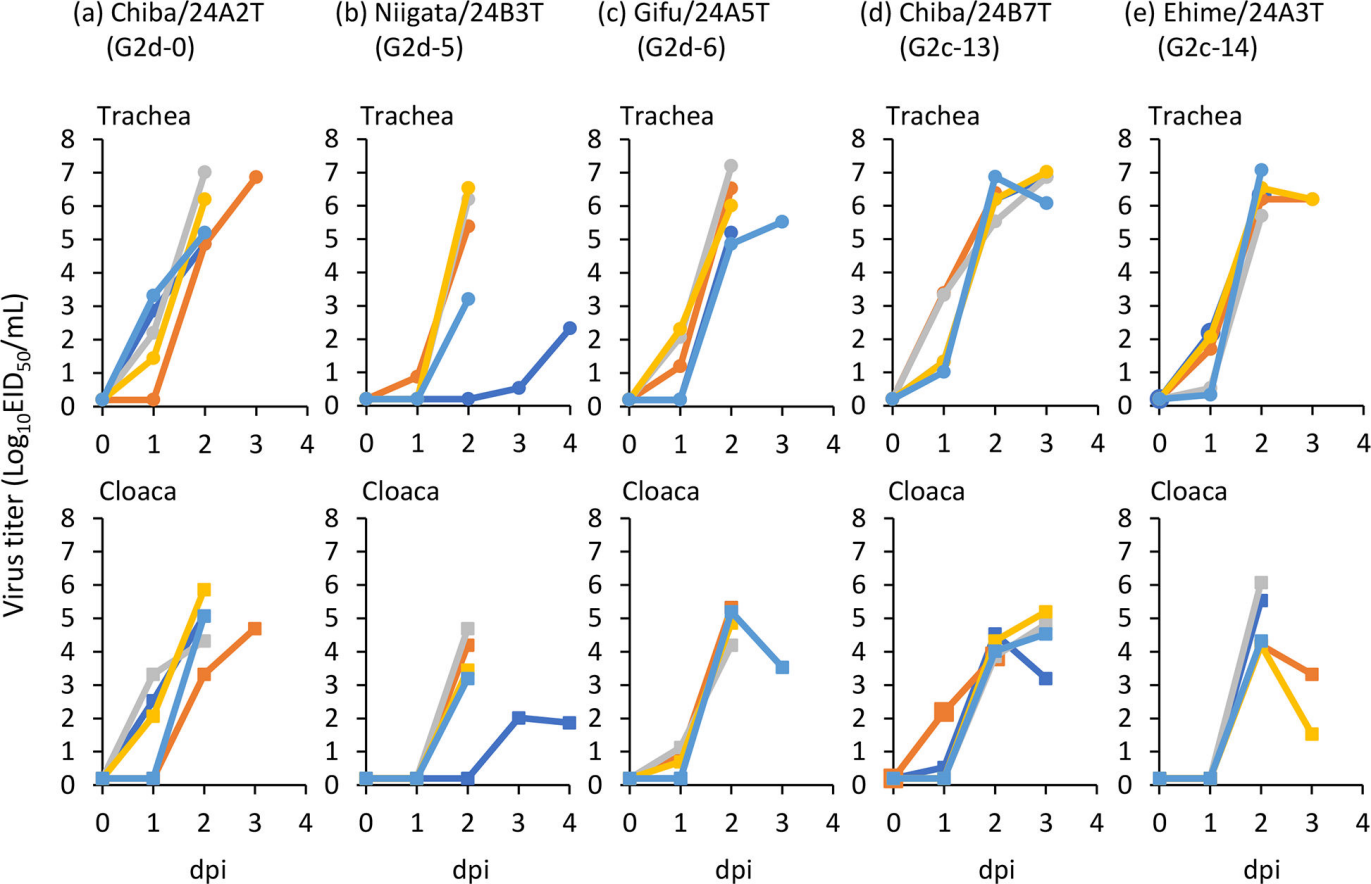
Viral titers in tracheal and cloacal swabs collected from chickens inoculated with 10^6^ 50% egg infectious dose (EID_50_) of representative virus strains. Viral titers in tracheal (circles) and cloacal (squares) swabs in chickens infected with representative virus strains: (**a**) Chiba/24A2T, (**b**) Niigata/24B3T, (**c**) Gifu/24A5T, (**d**) Chiba/24B7T, and (**e**) Ehime/24A3T. Colors indicate data from individual chickens: blue (#1), orange (#2), gray (#3), yellow (#4), and light blue (#5), corresponding to those shown in Fig. S3. dpi, days post-inoculation.

Viral titers peaked on the day of death or 1 day prior (Fig. 4). The maximum viral titers ranged from 4.73 to 6.80 log_10_ EID_50_/mL in tracheal samples and from 3.51 to 5.00 log_10_ EID_50_/mL in cloacal samples (Table 2), with tracheal titers generally exhibiting higher values. The AUC values were 522,972–5,025,084 for virus titers in tracheal samples, 7,047–161,279 for those in cloacal samples, and 530,019–5,066,470 for the sum of those from both sample types. Among the five strains identified during the 2024–2025 season, analysis of tracheal swabs showed that Chiba/24B7T exhibited the highest titers and AUC, and Niigata/24B3T exhibited the lowest values. The maximum viral titers and AUC varied among strains, although the differences were not statistically significant.

To compare the pathogenicity of HPAI viruses in the 2024–2025 season with those from previous seasons, CLD_50_, MDT, maximum viral titers, and AUC values of HPAI viruses in the 2022–2023 and 2023–2024 seasons were determined (Table S4). The CLD_50_ values of genotypes G2c-6, G2c-9, and G2c-12 tended to be higher than those of the G2c genotypes identified during the 2024–2025 season. The AUC values of Chiba/24A2T (G2d-0) and Gifu/24A5T (G2d-6) were high in the G2d subgroup (Fig. S4). The AUC values of Chiba/24B7T (G2c-13), Ehime/24A3T (G2c-14), and Aichi/22A3T (G2c-8) were high in the G2c subgroup, with Chiba/24B7T exhibiting the highest value. Differences in the AUC were not statistically significant.

### *In vitro* experiments using avian and bovine cells

**Fig 5:**
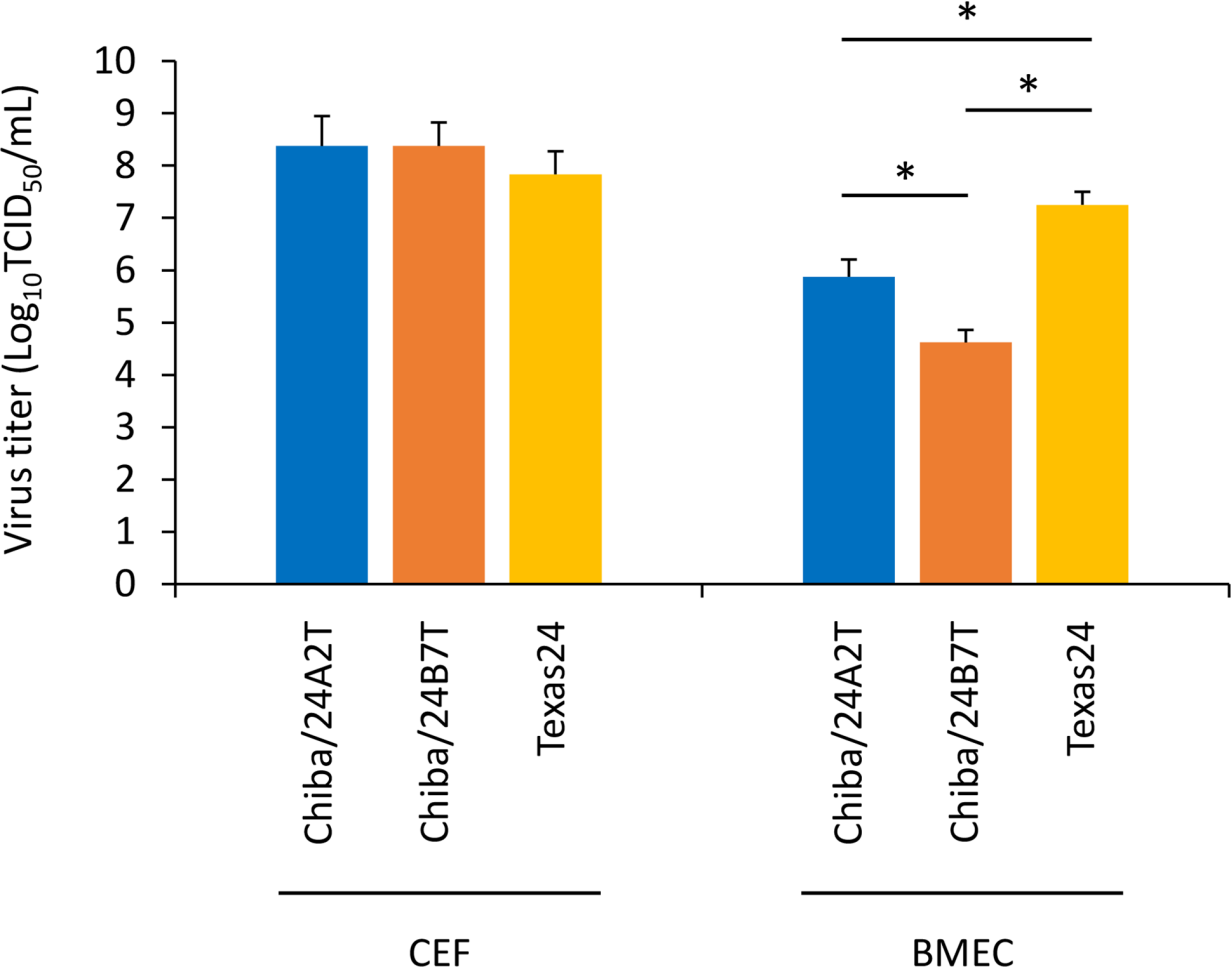
Infectivity of representative high pathogenicity avian influenza (HPAI) viruses in avian and bovine cells. Infectivity of representative virus strains in chicken embryo fibroblasts (CEFs) and bovine mammary epithelial cells (BMECs) was determined as the tissue culture infectious dose (TCID_50_)/mL. Values represent the mean values of three independent experiments. Asterisks indicate significant differences (*P* < 0.05) determined using one-way analysis of variance, followed by Tukey’s test.

To estimate the potential of the viruses isolated in Japan to infect dairy cattle, we compared the infectivity of the G2d-0 and G2c-13 genotypes with that of the B3.13 genotype in BMEC. The H5N1 HPAI virus of the genotype B3.13, Texas24 (37), was generated by reverse genetics. As shown in Fig. 5, viral infectivity is evaluated in both CEFs and BMECs, which are considered primary targets of the H5N1 HPAI viruses in cattle (40). Three viruses used in this study exhibited comparable infectivity in CEF, whereas the infectivity exhibited by Chiba/24A2T (G2d-0) and Chiba/24B7T (G2c-13) was at least 23-fold lower than that of Texas24 in BMECs.

## DISCUSSION

This study provides a comprehensive genomic overview of HPAI viruses circulating in Japan during the 2024–2025 season based on whole-genome sequencing of 273 isolates obtained from poultry and wild birds. *In vivo* and *in vitro* experiments were also conducted to determine the infectivity and pathogenicity of the representative strains isolated from chickens in Japan during the same season.

Eleven different genotypes (G2d-0, G2d-5–G2d-9, and G2c-13–G2c-17) were identified in Japan during the 2024–2025 season. Notably, two new genotypes (G2d-6 and G2d-8), resulting from reassortment between the G2c and G2d subgroups, were detected during this season, indicating ongoing genetic exchange within clade 2.3.4.4b. Such reassortment events can be interpreted within the geographic distributions of the two subgroups. Phylogenetic analyses conducted since the 2021–2022 season revealed that the G2d subgroup predominated along the North Pacific rim, including northern Japan, the Russian Far East, the Bering Sea region, Alaska, and the western coast of North America. Consistently, HPAI outbreaks in domestic poultry caused by the G2d subgroup historically occurred mainly in northern Japan during previous seasons (6, 7). However, G2d-related outbreaks had expanded across Japan since the 2023–2024 season, and viruses belonging to the G2d subgroup had begun to be reported in China and South Korea, suggesting expansion of the G2d subgroup into Asian countries via Japan and surrounding regions.

Japanese G2c subgroup isolates formed monophyletic clusters with isolates mainly reported in Asian countries. Genetic analyses of viruses from the 2022–2023 season implied that diverse G2c viruses were likely to have been introduced into Japan via the Korean Peninsula or China and Russia, mainly crossing the Sea of Japan directly (7). Recent studies from China have reported seven genotypes and propose that some of them are transmitted from northern China to Japan (41). Indeed, genotypes N1.1 and N1.2 within the Chinese GII group correspond to G2c-8 and G2c-2 in Japan, respectively (7). The detection of nearly identical G2c viruses in both countries indicated that the G2c subgroup had repeatedly circulated between Japan and other Asian countries since at least 2022–2023. Given these distinct geographic distributions, Japan and neighboring regions likely acted as ecological relay points where migratory birds from different flyways intersected (42, 43), facilitating the dynamic movement and mixing of viral populations, as evidenced by the concurrent detection of G2c and G2d subgroups in Japan during the same seasons and the emergence of their reassortment viruses.

We conducted *in vivo* experiments using chickens to determine whether genotype differences affect viral characteristics, as outbreak patterns differed substantially among genotypes during the 2024–2025 season (Table 1). Total viral shedding from chickens was quantified using the AUC (33, 34). Chickens infected with Niigata/24B3T (G2d-5) had markedly lower AUC values than those infected with the other two G2d genotypes (Table 2; Fig. S4), whereas Chiba/24B7T (G2c-13) showed the highest average AUC values within the G2c subgroup, including genotypes from previous seasons (Table 2; Table S4; Fig. S4). Geographically, the majority of G2d-0 (13 out of 22) and G2c-13 (15 out of 24) outbreaks occurred in areas with a high concentration of poultry farms in Aichi and Chiba prefectures (Fig. S2). In contrast, no significant difference in CLD_50_ was observed among the five genotypes (Table 2), suggesting no obvious correlation between CLD_50_ and outbreak frequency. Considering these factors, the incursion of viruses exhibiting relatively large-scale viral shedding in livestock-dense regions may have contributed to consecutive outbreaks during the 2024–2025 season.

Genotypes G2c-13 and G2d-0 were shown to be responsible for major outbreaks in poultry farms in Japan even if consecutive outbreaks in Aichi and Chiba prefectures were excluded. Accordingly, genotype G2d-0 was detected most frequently through surveillance of dead wild birds (Table 1). However, the number of G2c-13 detections in dead wild birds tended to be remarkably lower than that of outbreaks in poultry farms. Therefore, new strategies, including actively surveying live birds and/or environmental water sources, are required to address the discrepancy between outbreaks in poultry farms and wild bird surveillance.

The G2c-6, G2c-9, G2c-12, and G2d-5 genotypes, which exhibited substantially lower viral shedding than the other five genotypes (G2c-8, G2c-13, G2c-14, G2d-0, and G2d-6), possess methionine at the position 105 in the NP (Table 2; Table S4 and S5). In contrast, the other five genotypes (G2c-8, G2c-13, G2c-14, G2d-0, and G2d-6) possess valine at the same position of the NP (Table S5). This mutation (M105V) has been reported to be responsible for increased pathogenicity in chickens (44, 45). Thus, it may have been associated with varying levels of viral shedding among the individual genotypes examined in the present study. Further study is necessary to ascertain the contribution of M105V to viral shedding from chickens infected with different genotypes.

Chiba/24A2T (G2d-0) and Chiba/24B7T (G2c-13) exhibited markedly lower infectivity in BMECs than Texas24 (B3.13), which was responsible for major outbreaks in dairy cattle in the United States (Fig. 5). This suggested that the current risk posed by these Japanese virus strains infecting dairy cattle was considerably lower than that posed by Texas24. However, this comparison between two representative virus strains in Japan and Texas24 did not comprehensively reflect the risk of spillover to dairy cattle, as it is believed that Texas24 has already adapted to dairy cattle. Recent genomic studies have reported that the M631L mutation in PB2 is frequently detected in viruses belonging to genotype B3.13 (14). This mutation is highly likely to be an adaptive mutation to cattle (46); however, it was not detected in viruses isolated in Japan, supporting the interpretation that the risk of transmission to dairy cattle remained low. In addition to genotype B3.13, genotype D1.1 caused outbreaks in dairy cattle in the United States. The genotype D1.1 transmitted to dairy cattle does not have the M631L mutation (14, 16), but has the E627K and D701N mutations in PB2, which are associated with mammalian adaptation of HPAI viruses (44). Genotype G2d-0 shares the HA and three other internal (PB1, MP, and NS) genes with genotype D1.1 (13, 17). The HPAI viruses in Japan during the 2024–2025 season did not have the E627K and D701N mutations, suggesting that these viruses have limited potential for transmission to cattle. However, the E627K and D701N mutations were identified in several HPAI viruses isolated in Japan during the 2022–2023 season (7). The susceptibility of BMECs to these viruses warrants further investigation.

In summary, our findings highlight the extensive genetic diversity and ongoing reassortment among clade 2.3.4.4b H5N1 viruses circulating in Japan during the 2024–2025 season. Despite similar lethality across genotypes in chickens, viruses exhibiting relatively substantial viral shedding may have contributed to consecutive outbreaks in regions with high poultry density. The markedly lower infectivity of genotypes G2d-0 and G2c-13 in BMECs compared with that of genotype B3.13 indicated a limited risk of spillover to dairy cattle in Japan. These findings highlight the importance of continued attention to the epidemiological and biological properties of the G2 group for developing effective prevention strategies in Japan.

## Data Availability

Sequence data obtained in this study have been deposited in the GISAID database. Isolate IDs are listed in Tables S1 and S2.

## SUPPLEMENTAL MATERIAL

Tables S1 to S5 spectrum.00293-26-s0001.docx Information of each isolate derived from poultry and wild birds, information of gene segments closely related with each HPAI virus, virological characteristics of H5N1 viruses in 2022-2023 and 2023-2024, and list of amino acid mutations important for pathogenicity.

Figures S1 to S4 spectrum.00293-26-s0002.pdf Phylogenetic trees of each gene segment, geographic distribution of HPAI cases in Japan, history of chickens inoculated with each HPAI strain, and the comparison of area under the curve of viral titer detected from virus-inoculated chickens.

ASM does not own the copyrights to Supplemental Material that may be linked to, or accessed through, an article. The authors have granted ASM a non-exclusive, world-wide license to publish the Supplemental Material files. Please contact the corresponding author directly for reuse.
