# Supplementary material for: High pathogenicity avian influenza A (H5N1) viruses isolated from poultry and wild birds in Japan during the 2024–2025 season
Source: Microbiol Spectr. 2026 Jul 14;14(8):e00293-26. doi: 10.1128/spectrum.00293-26 (PMC13435830; doi:10.1128/spectrum.00293-26)
Supplement: Supplementary file 1 [file spectrum.00293-26-s0002.pdf]

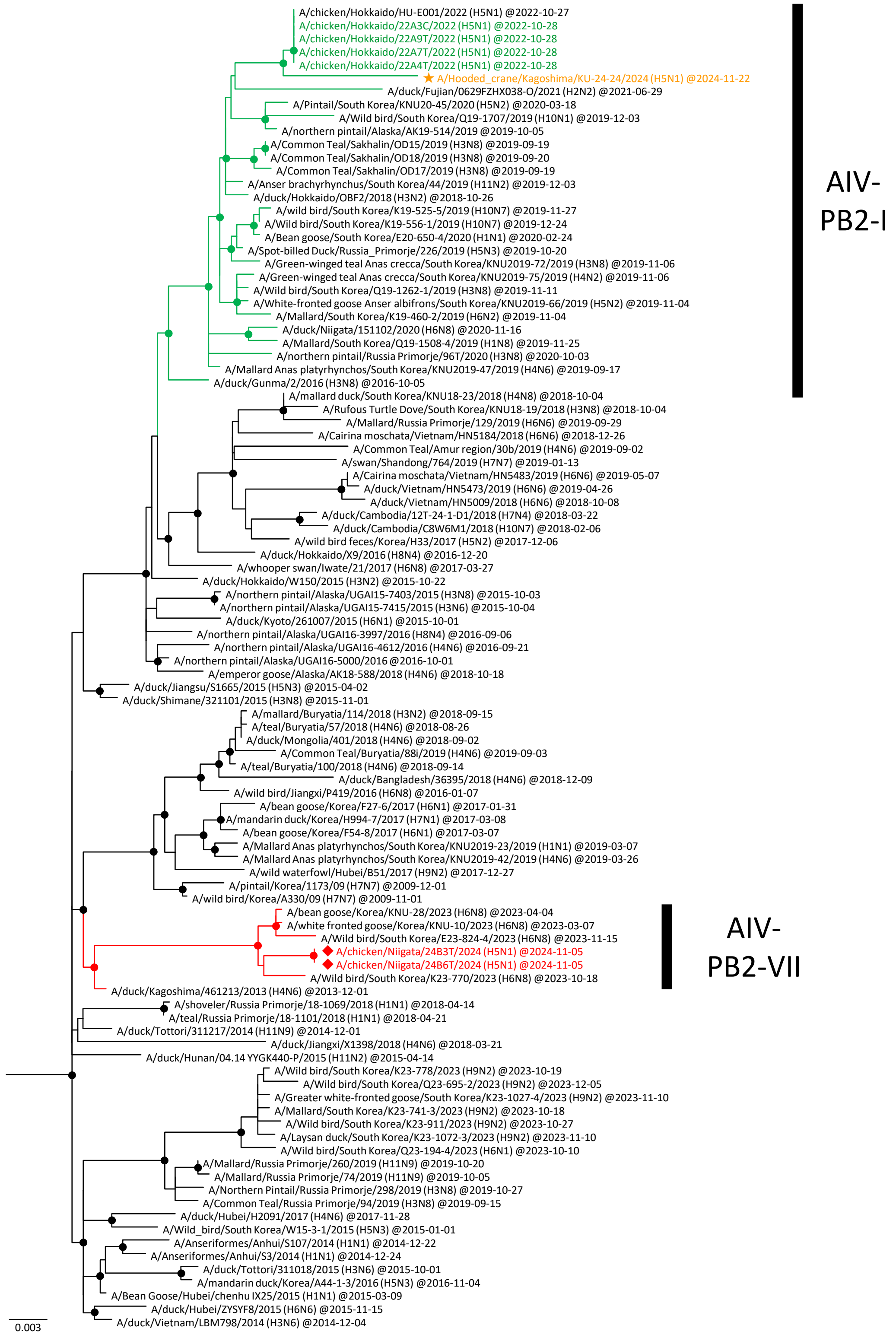

Fig. S1a. Partial polymerase basic protein 2 (PB2) gene phylogenic tree showing the AIV-PB2-VII cluster.

Isolates marked with a red diamond (◆) represent isolates derived from Japanese poultry and analyzed at the National Institute of Animal Health (NIAH) in the 2024/25 season. Those marked with a yellow star (★) represent isolates derived from wild birds collected in Japan during the same season, and those shown in green represent isolates from Japanese poultry analyzed at the NIAH in the 2022/23 season, which belong to the AIV-NP-IV cluster (Takadate et al., 2024). Filled circles (●) at nodes indicate Shimodaira–Hasegawa approximate likelihood ratio test (SH-aLRT)/bootstrap support values of ≥80/95.

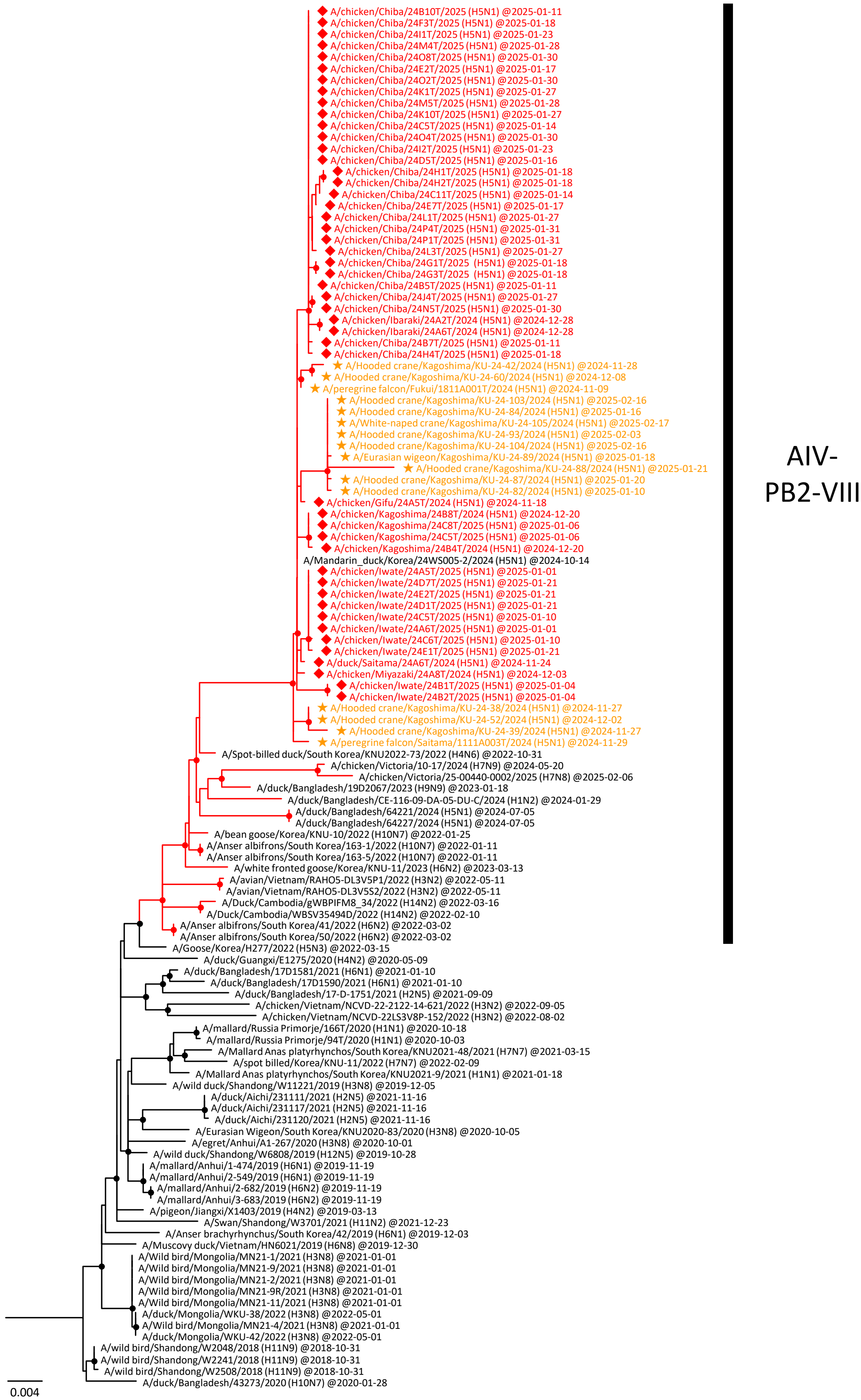

Fig. S1b. Partial polymerase basic protein 2 (PB2) gene phylogenic tree showing the AIV-PB2-VIII cluster.

Isolates marked with a red diamond (◆) represent isolates derived from Japanese poultry and analyzed at the National Institute of Animal Health (NIAH) in the 2024/25 season, and those marked with a yellow star (★) represent isolates derived from wild birds collected in Japan during the same season. Filled circles (●) at nodes indicate Shimodaira–Hasegawa approximate likelihood ratio test (SH-aLRT)/bootstrap support values of ≥80/95**.**

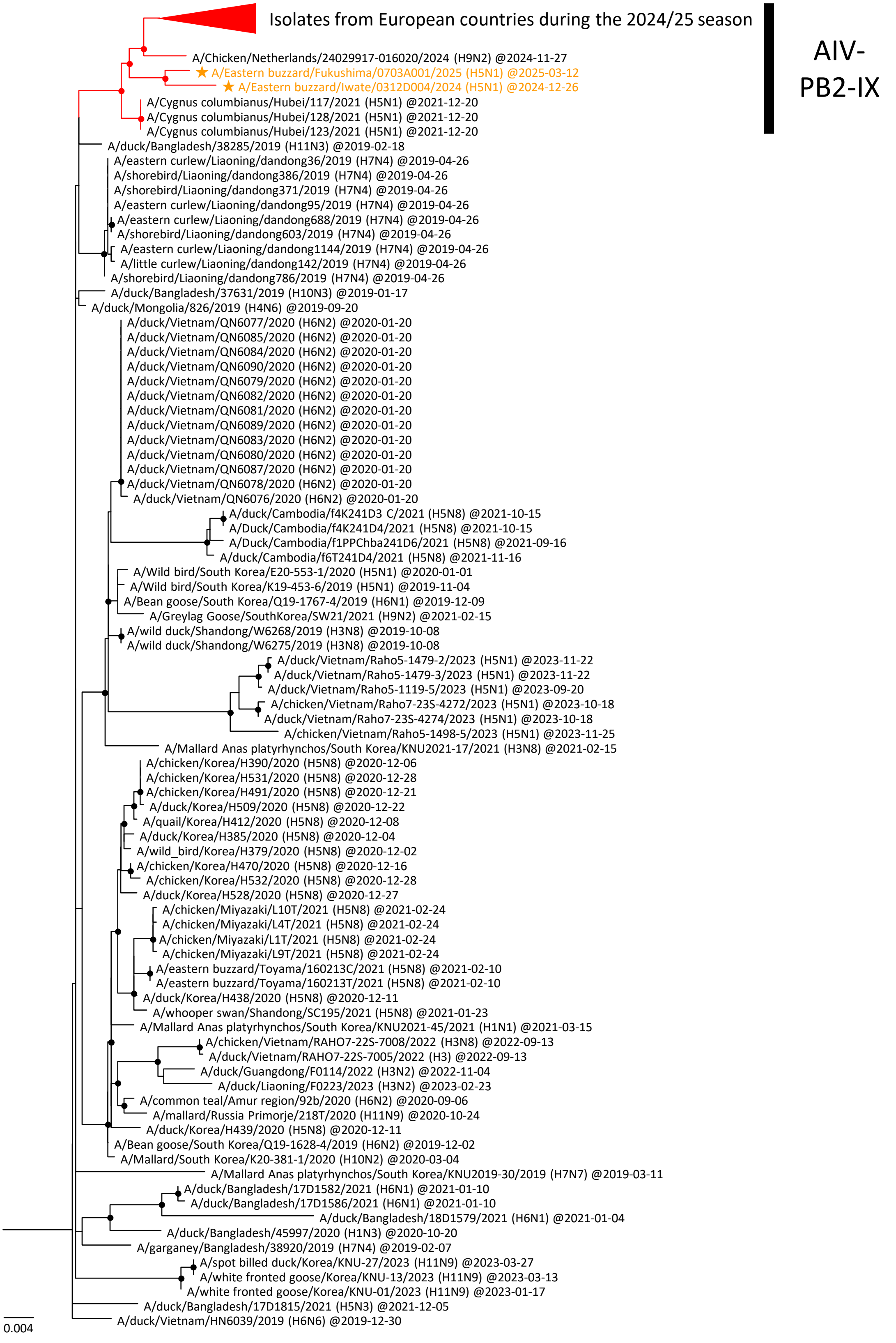

Fig. S1c. Partial polymerase basic protein 2 (PB2) gene phylogenic tree showing the AIV-PB2-IX cluster.

Isolates marked with a red diamond (◆) represent isolates derived from Japanese poultry and analyzed at the National Institute of Animal Health (NIAH) in the 2024/25 season, and those marked with a yellow star (★) represent isolates derived from wild birds collected in Japan during the same season. Filled circles (●) at nodes indicate Shimodaira–Hasegawa approximate likelihood ratio test (SH-aLRT)/bootstrap support values of ≥80/95.

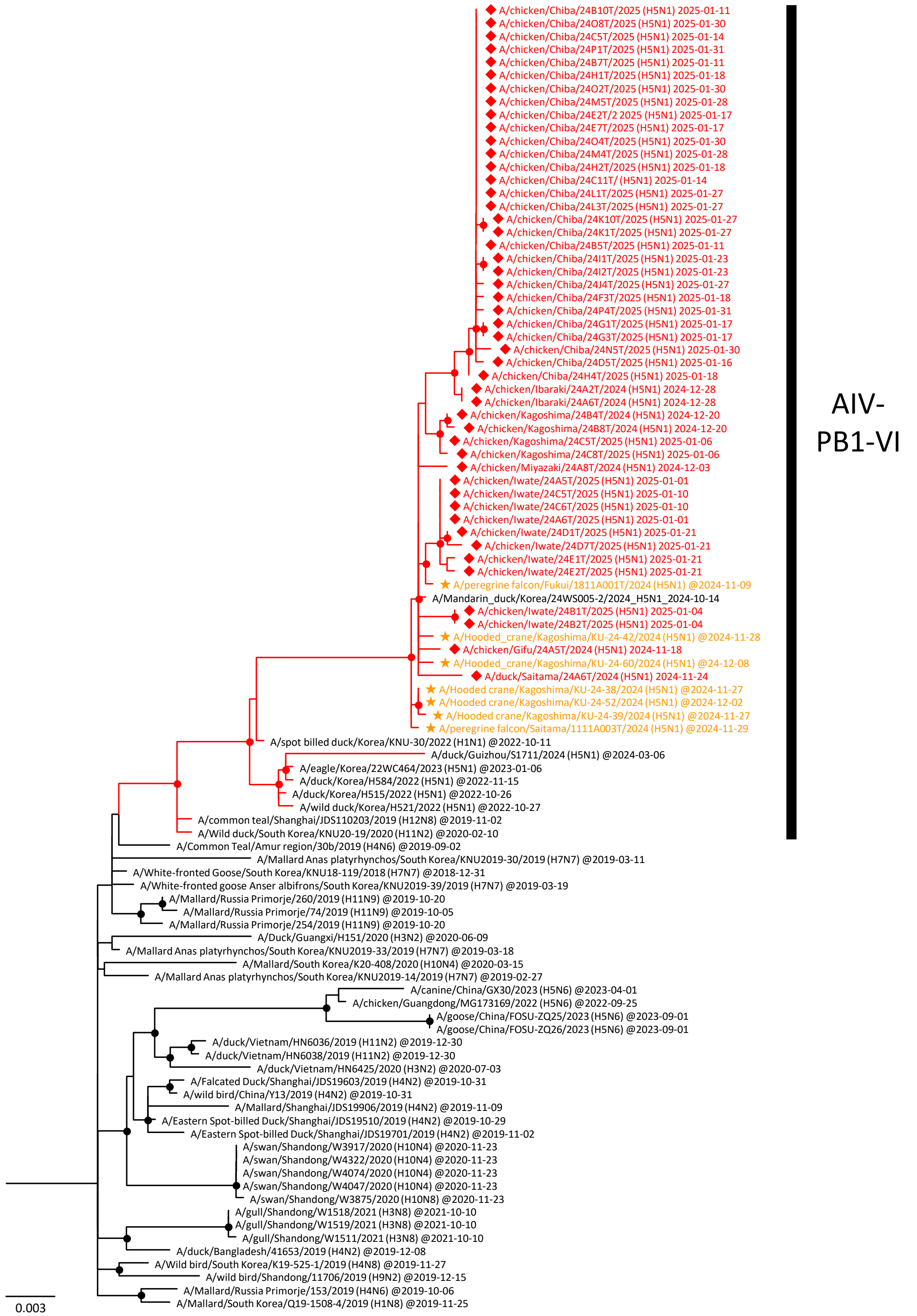

Fig. S1d. Partial polymerase basic protein 1 (PB1) gene phylogenic tree showing the AIV-PB1-VI cluster.

Isolates marked with a red diamond (◆) represent isolates derived from Japanese poultry and analyzed at the National Institute of Animal Health (NIAH) in the 2024/25 season, and those marked with a yellow star (★) represent isolates derived from wild birds collected in Japan during the same season. Filled circles (●) at nodes indicate Shimodaira–Hasegawa approximate likelihood ratio test (SH-aLRT)/bootstrap support values of ≥80/95.

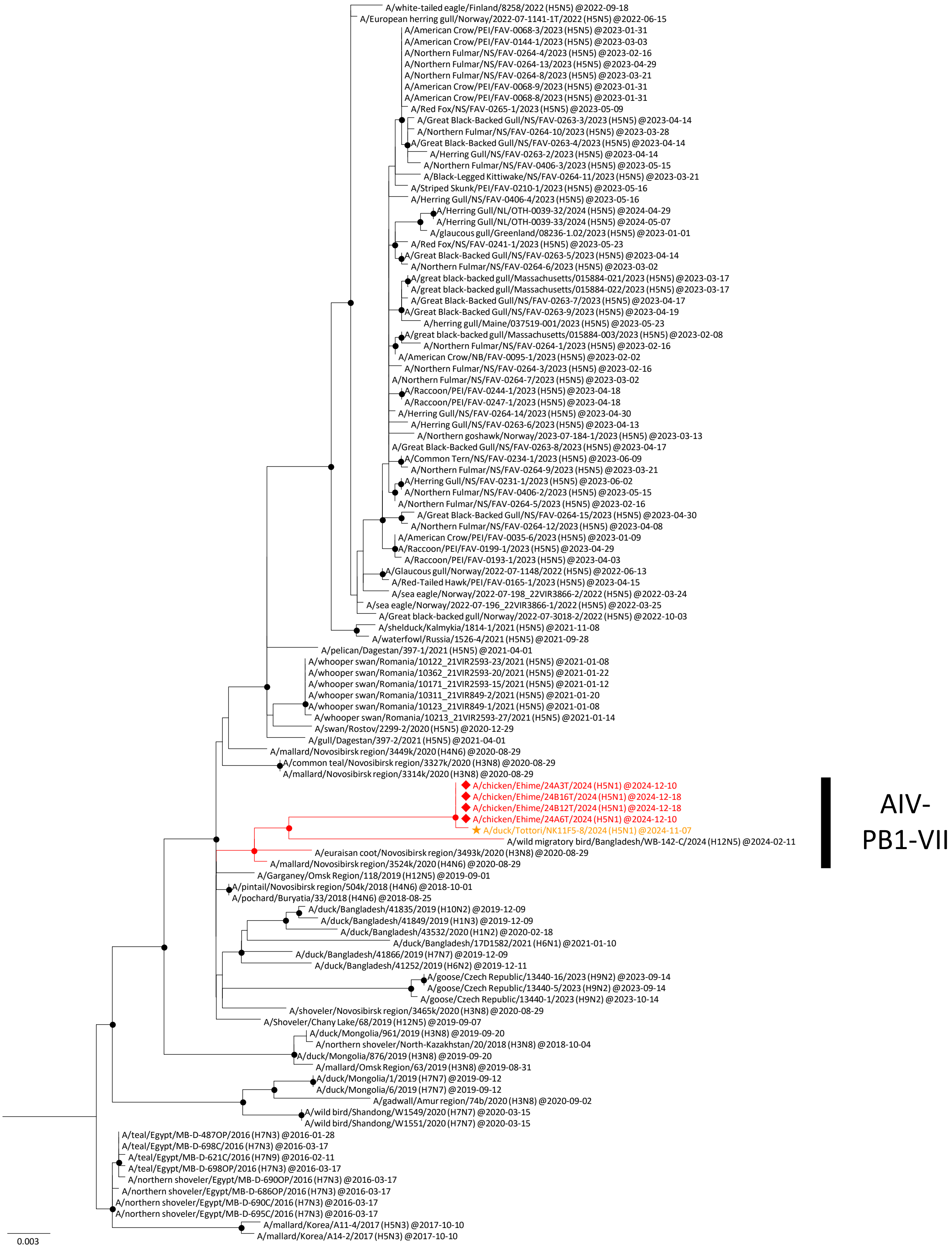

Fig. S1e. Partial polymerase basic protein 1 (PB1) gene phylogenic tree showing the AIV-PB1-VII cluster. Isolates marked with a red diamond (◆) represent isolates derived from Japanese poultry and analyzed at the National Institute of Animal Health (NIAH) in the 2024/25 season, and those marked with a yellow star (★) represent isolates derived from wild birds collected in Japan during the same season. Filled circles (●) at nodes indicate Shimodaira–Hasegawa approximate likelihood ratio test (SH-aLRT)/bootstrap support values of ≥80/95.

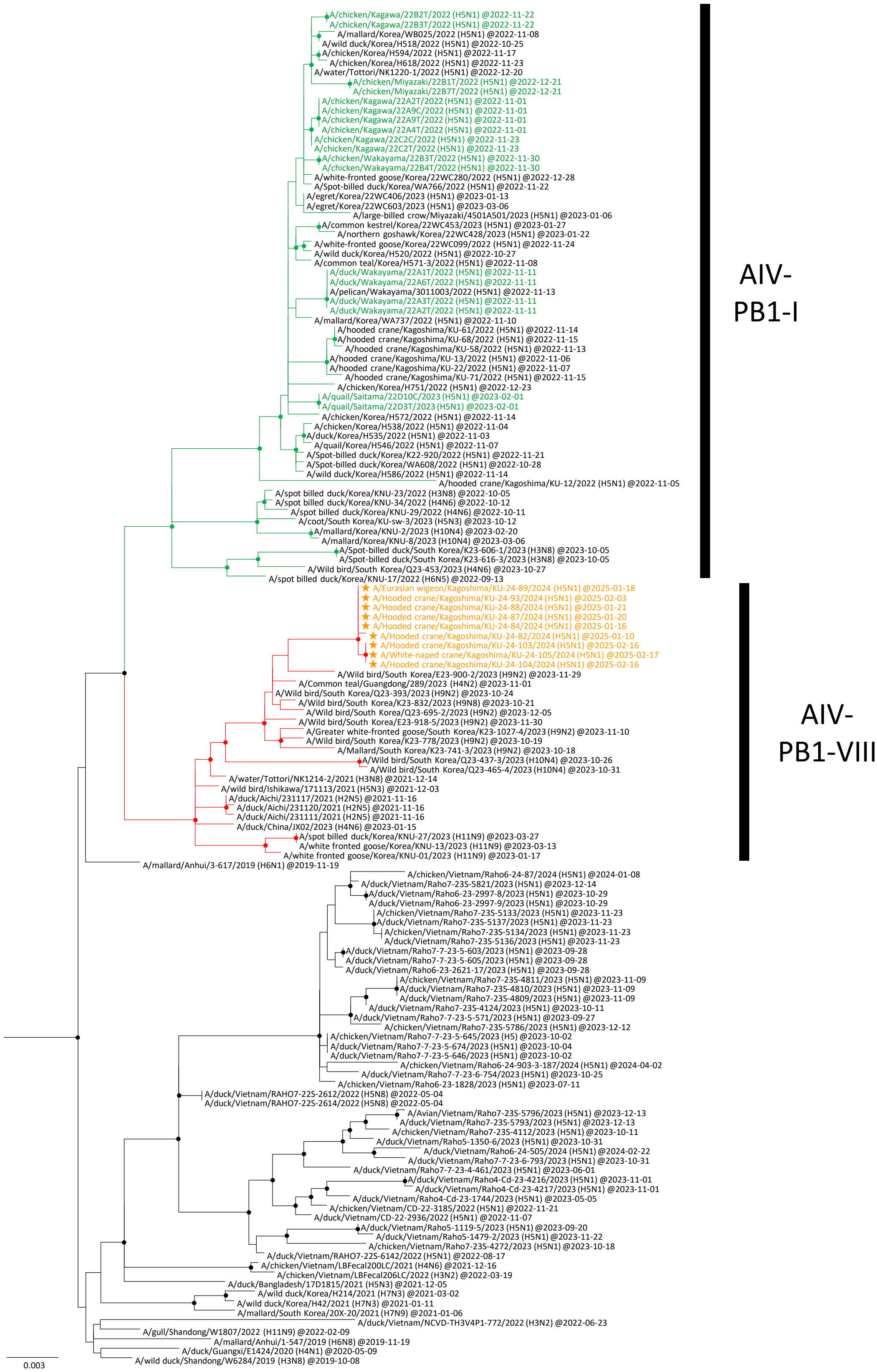

Fig. S1f. Partial polymerase basic protein 1 (PB1) gene phylogenic tree showing the AIV-PB1-VIII cluster. Isolates marked with a yellow star (★) represent isolates derived from wild birds collected in Japan during the 2024/25 season, and those shown in green represent isolates derived from Japanese poultry and analyzed at the National Institute of Animal Health (NIAH) in the 2022/23 season, which belong to the AIV-NP-IV cluster (Takadate et al., 2024). Filled circles (●) at nodes indicate Shimodaira–Hasegawa approximate likelihood ratio test (SH-aLRT)/bootstrap support values of ≥80/95.

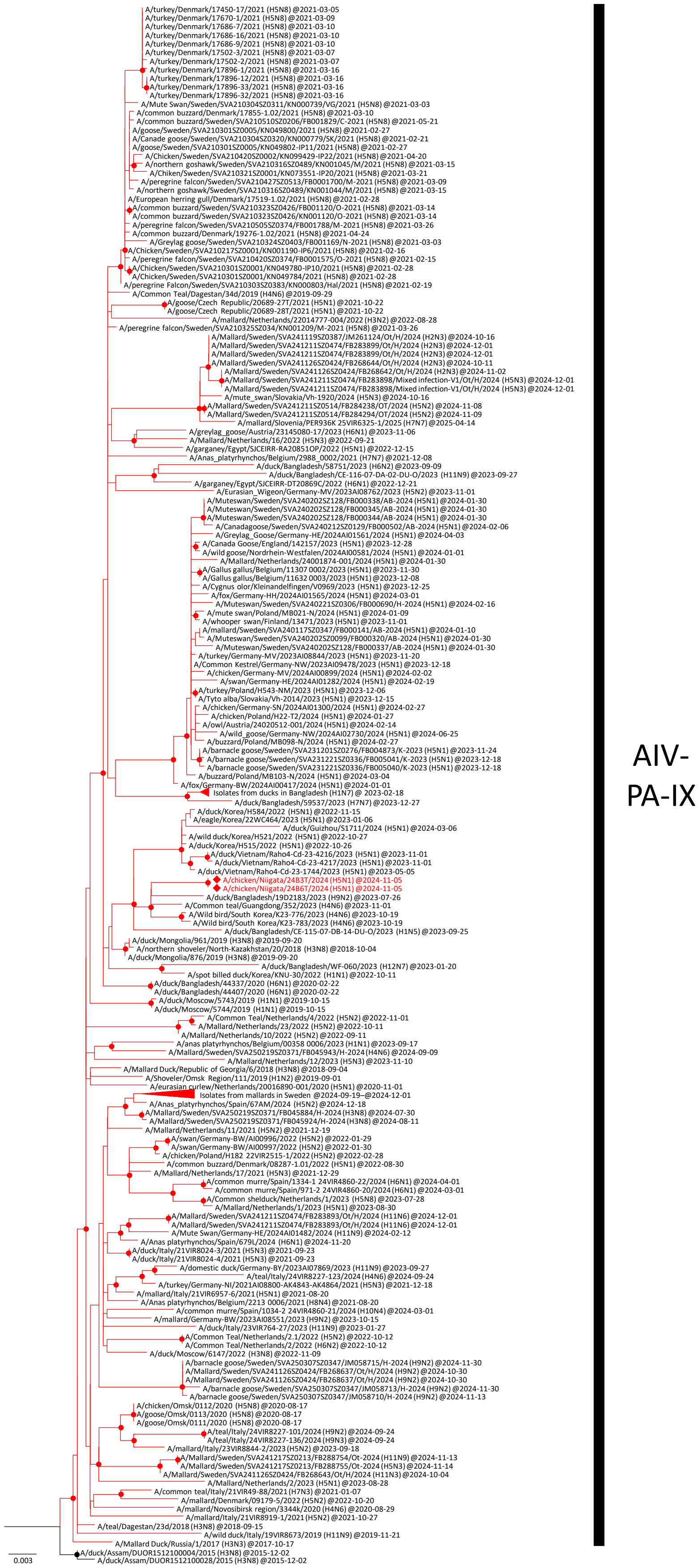

Fig. S1g. Partial polymerase acidic protein (PA) gene phylogenic tree showing the AIV-PA-IX cluster.

Isolates marked with a red diamond (◆) represent isolates derived from Japanese poultry and analyzed at the National Institute of Animal Health (NIAH) in the 2024/25 season. Filled circles (●) at nodes indicate Shimodaira–Hasegawa approximate likelihood ratio test (SH-aLRT)/bootstrap support values of ≥80/95.

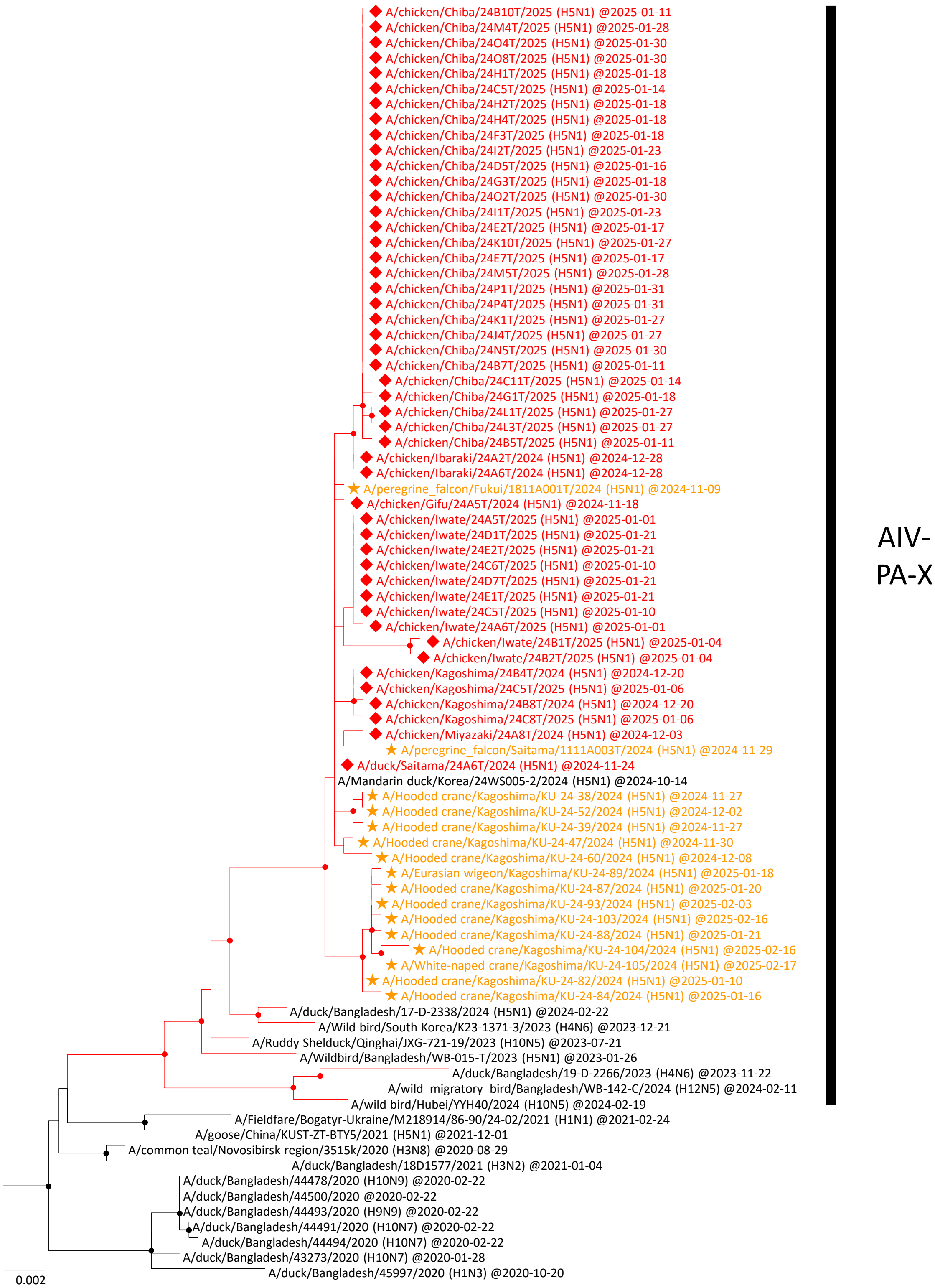

Fig. S1h. Partial polymerase acidic protein (PA) gene phylogenic tree showing the AIV-PB1-X cluster.

Isolates marked with a red diamond (◆) represent isolates derived from Japanese poultry and analyzed at the National Institute of Animal Health (NIAH) in the 2024/25 season, and those marked with a yellow star (★) represent isolates derived from wild birds collected in Japan during the same season. Filled circles (●) at nodes indicate Shimodaira–Hasegawa approximate likelihood ratio test (SH-aLRT)/bootstrap support values of ≥80/95.

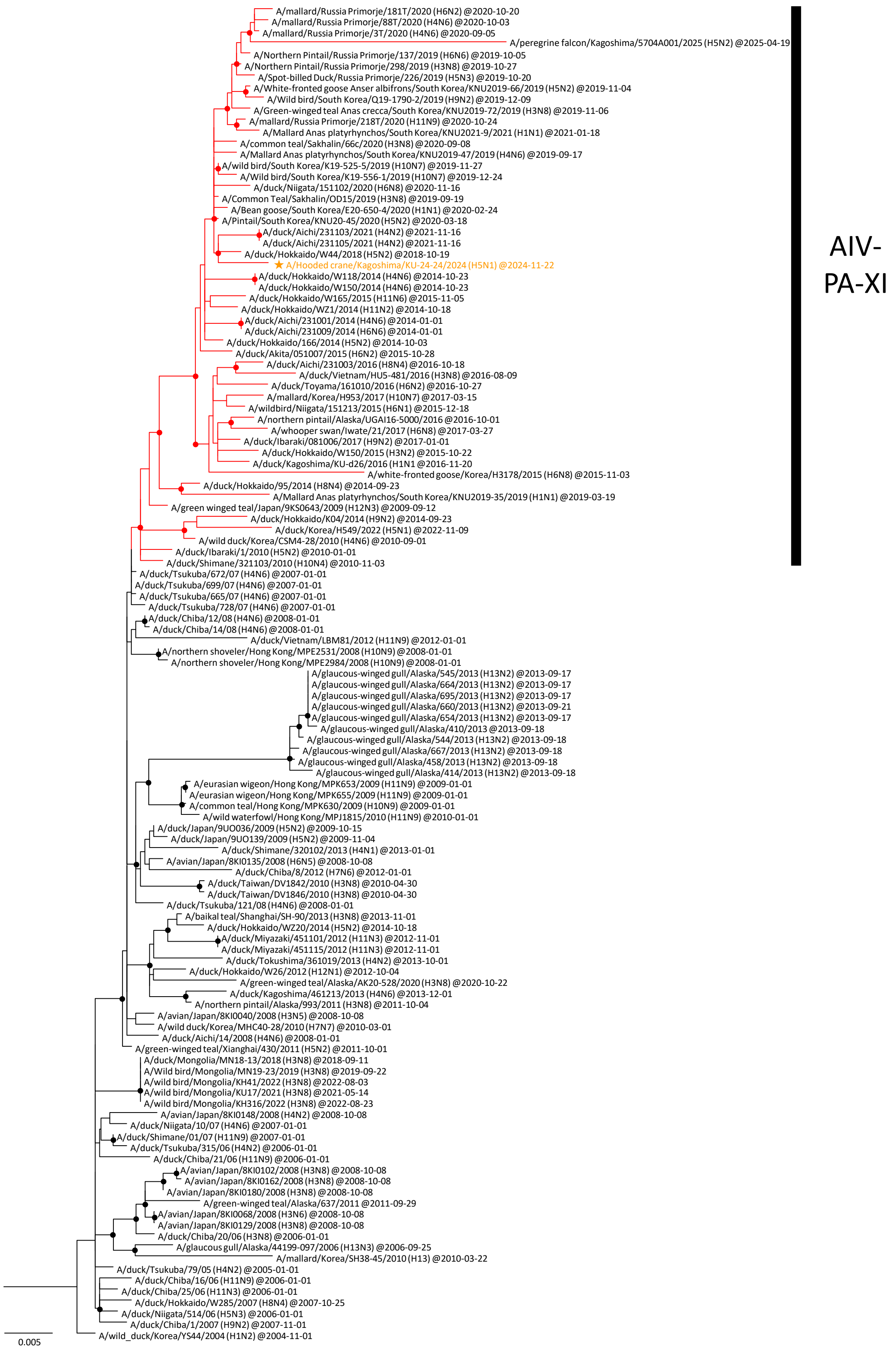

Fig. S1i. Partial polymerase acidic protein (PA) gene phylogenic tree showing the AIV-PB1-XI cluster.

Isolates marked with a yellow star (★) represent isolates derived from wild birds collected in Japan during the 2024/25 season. Filled circles (●) at nodes indicate Shimodaira–Hasegawa approximate likelihood ratio test (SH-aLRT)/bootstrap support values of ≥80/95.

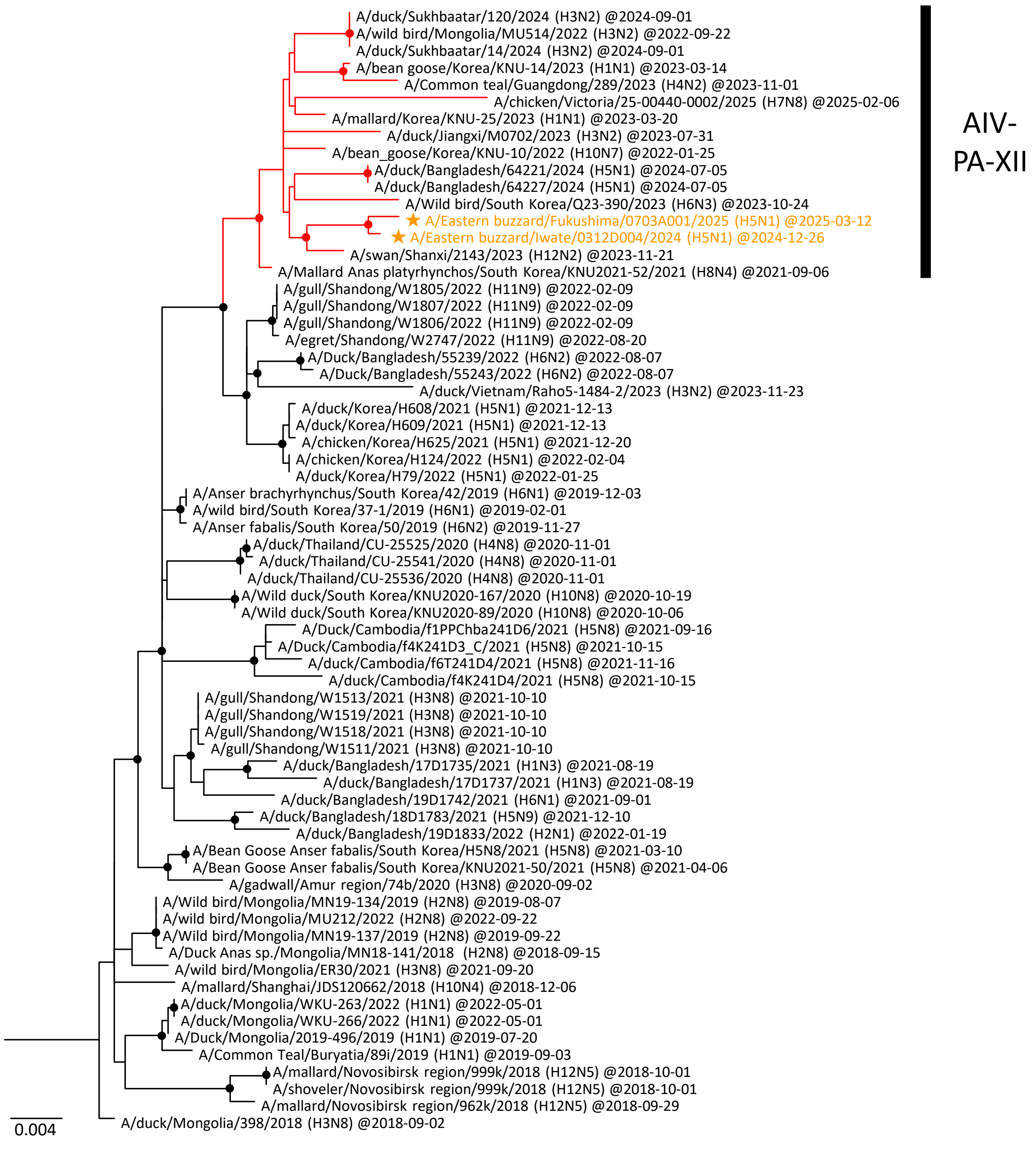

Fig. S1j. Partial polymerase acidic protein (PA) gene phylogenic tree showing the AIV-PB1-XII cluster.

Isolates marked with a yellow star (★) represent isolates derived from wild birds collected in Japan during the 2024/25 season. Filled circles (●) at nodes indicate Shimodaira–Hasegawa approximate likelihood ratio test (SH-aLRT)/bootstrap support values of ≥80/95.

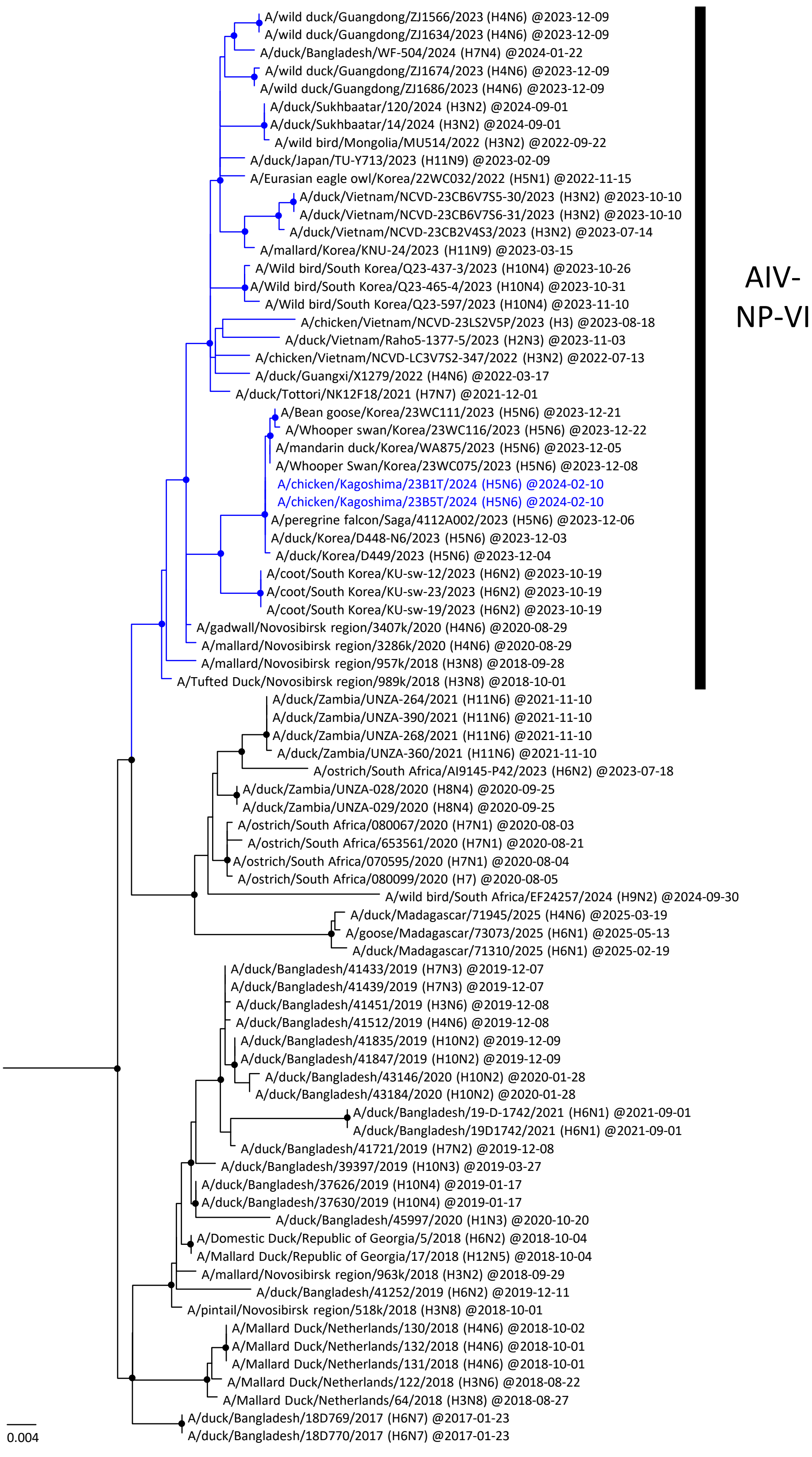

Fig. S1k. Partial nucleoprotein (NP) gene phylogenic tree showing the AIV-NP-VI cluster.

Isolates shown in blue represent isolates derived from Japanese poultry and analyzed at the National Institute of Animal Health (NIAH) in the 2023/24 season. Filled circles (●) at nodes indicate Shimodaira–Hasegawa approximate likelihood ratio test (SH-aLRT)/bootstrap support values of ≥80/95.

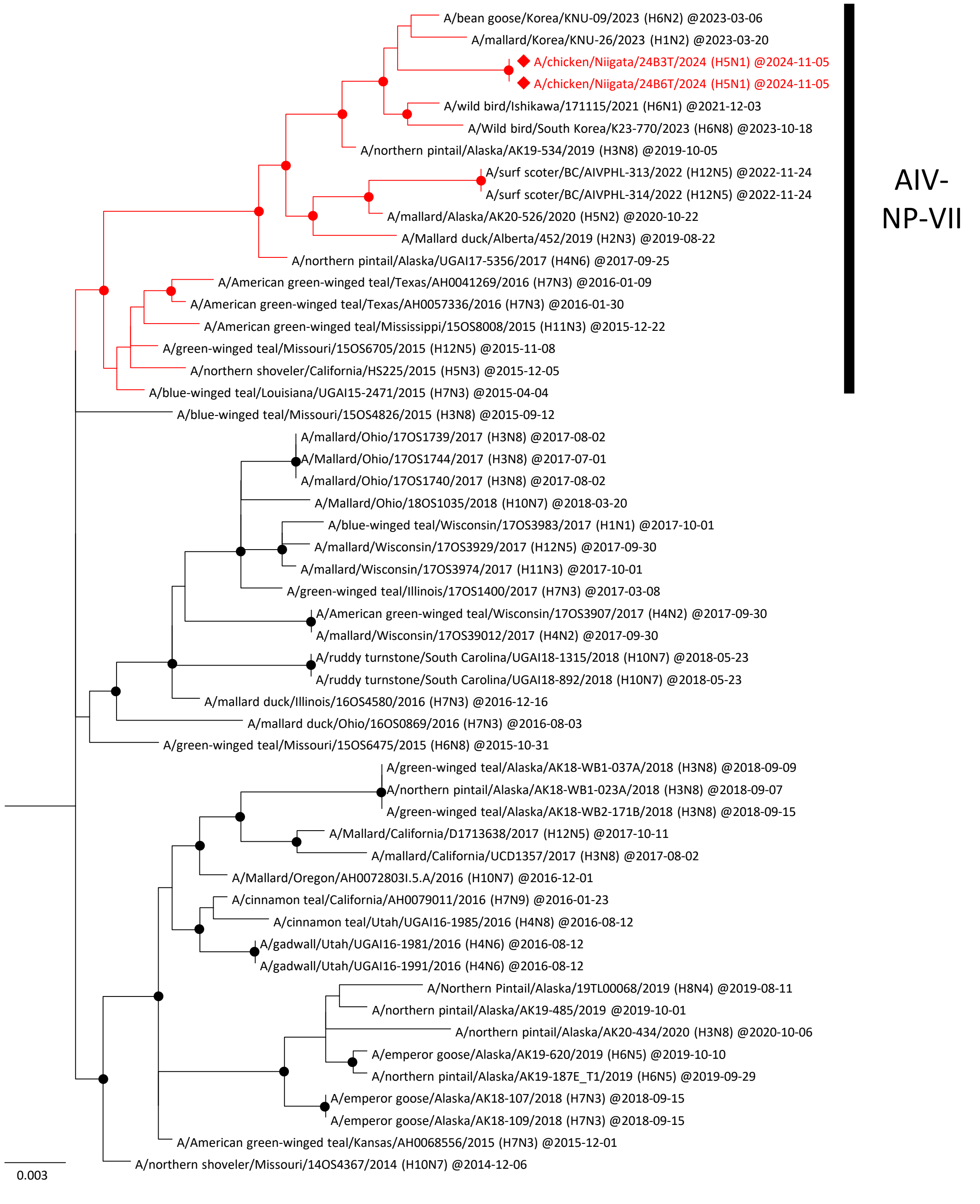

Fig. S1l. Partial nucleoprotein (NP) gene phylogenic tree showing the AIV-NP-VII cluster. Isolates marked with a red diamond (◆) represent isolates derived from Japanese poultry and analyzed at the National Institute of Animal Health (NIAH) in the 2024/25 season. Filled circles (●) at nodes indicate Shimodaira–Hasegawa approximate likelihood ratio test (SH-aLRT)/bootstrap support values of ≥80/95.

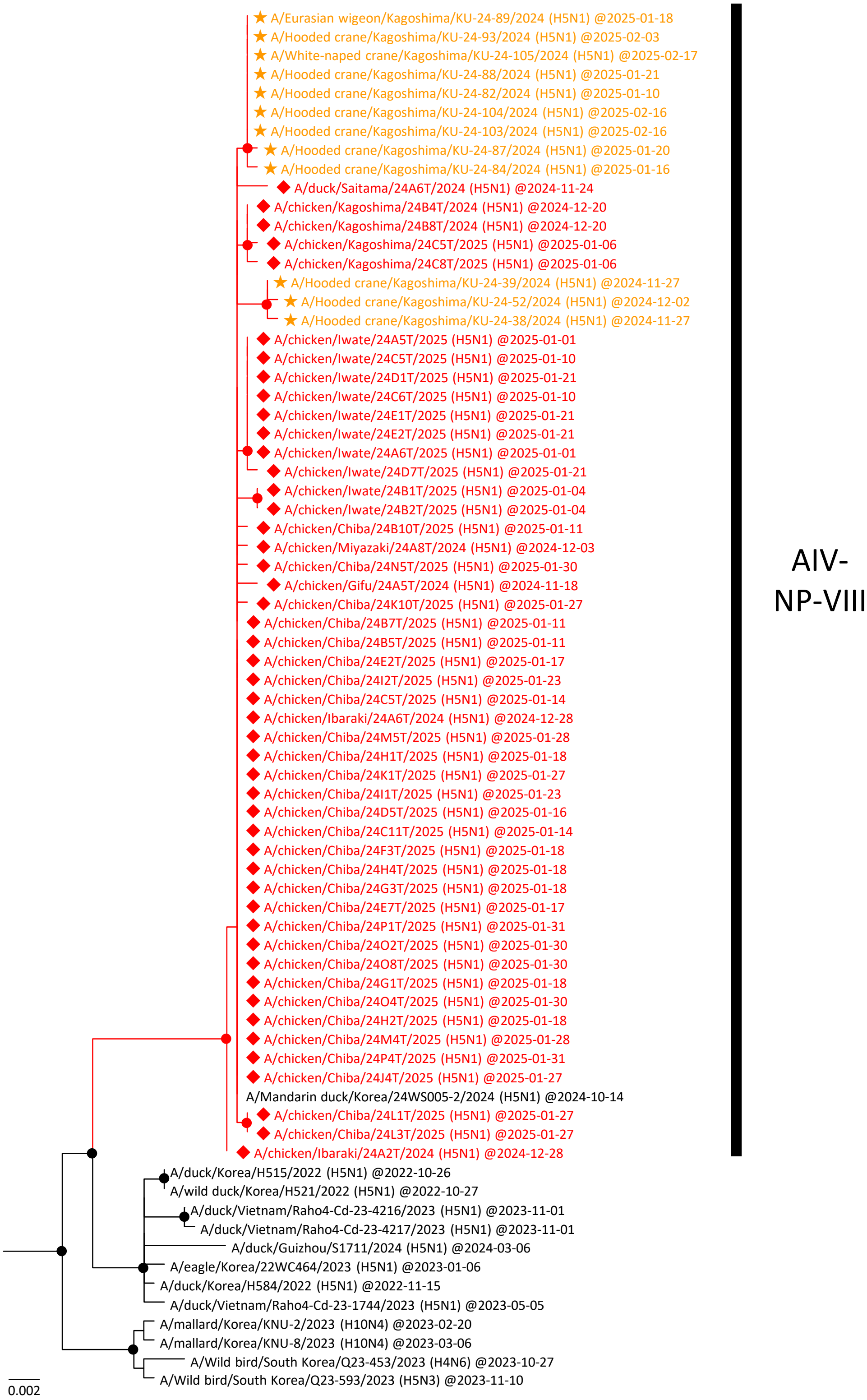

Fig. S1m. Partial nucleoprotein (NP) gene phylogenic tree showing the AIV-NP-VIII cluster.

Isolates marked with a red diamond (◆) represent isolates derived from Japanese poultry and analyzed at the National Institute of Animal Health (NIAH) in the 2024/25 season, and those marked with a yellow star (★) represent isolates derived from wild birds collected in Japan during the same season. Filled circles (●) at nodes indicate Shimodaira–Hasegawa approximate likelihood ratio test (SH-aLRT)/bootstrap support values of ≥80/95.

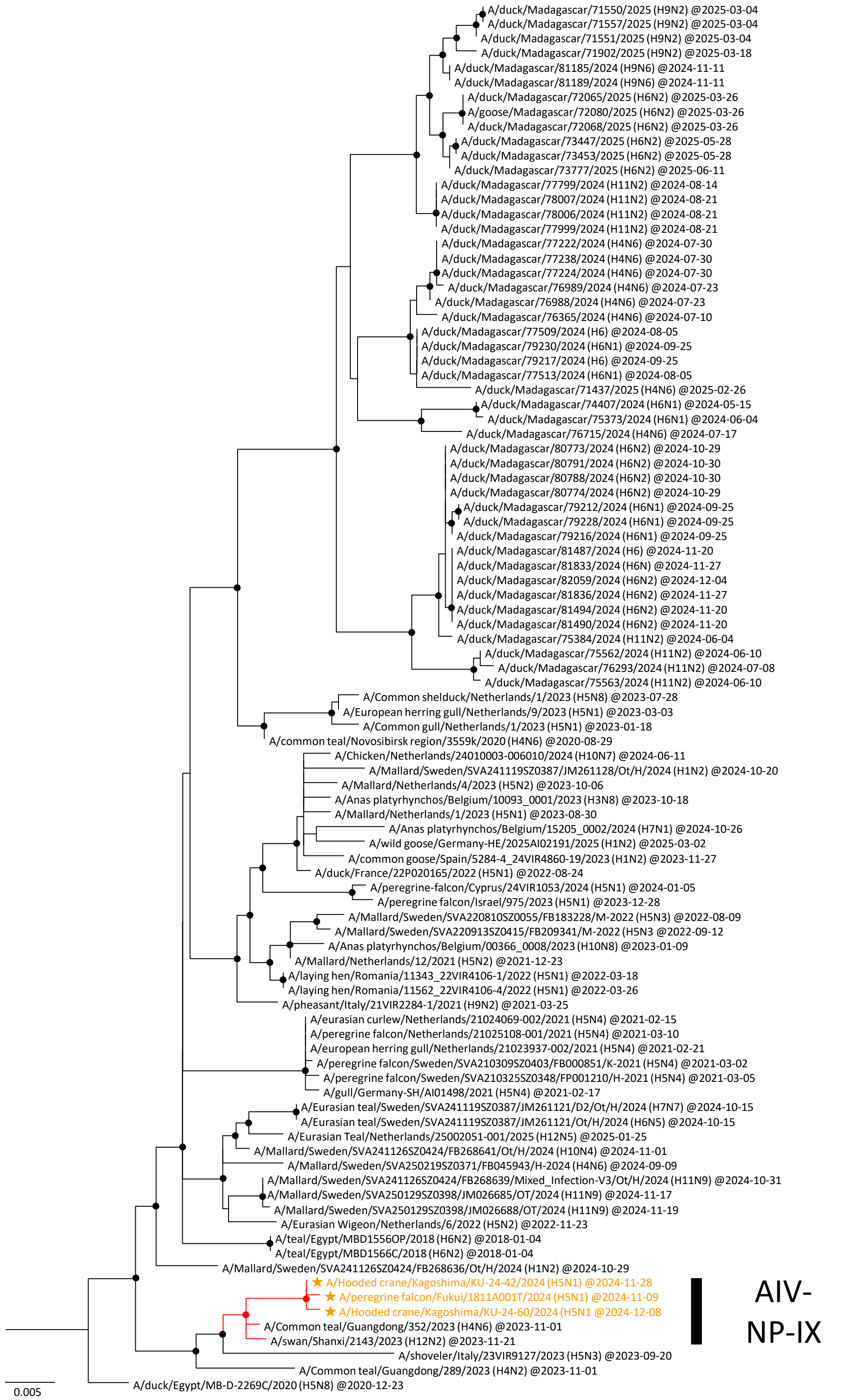

Fig. S1n. Partial nucleoprotein (NP) gene phylogenic tree showing the AIV-NP-IX cluster. Isolates marked with a yellow star (★) represent isolates derived from wild birds collected in Japan during the 2024/25 season. Filled circles (●) at nodes indicate Shimodaira–Hasegawa approximate likelihood ratio test (SH-aLRT)/bootstrap support values of ≥80/95.

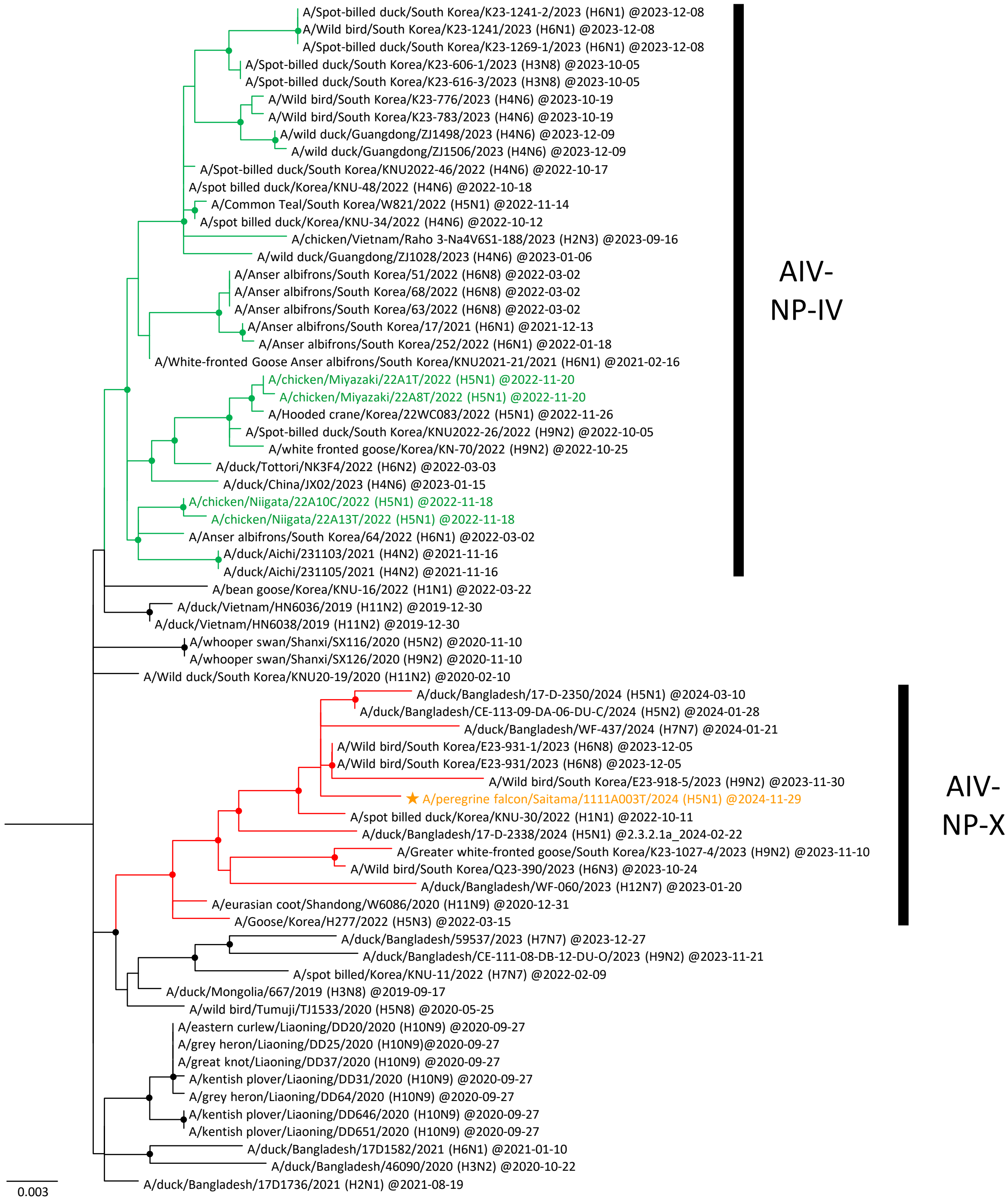

Fig. S1o. Partial nucleoprotein (NP) gene phylogenic tree showing the AIV-NP-X cluster.

Isolates marked with a yellow star (★) represent isolates derived from wild birds collected in Japan during the 2024/25 season, and isolates shown in green represent isolates derived from Japanese poultry and analyzed at the National Institute of Animal Health (NIAH) in the 2022/23 season, which belong to the AIV-NP-IV cluster (Takadate et al., 2024). Filled circles (●) at nodes indicate Shimodaira–Hasegawa approximate likelihood ratio test (SH-aLRT)/bootstrap support values of ≥80/95.

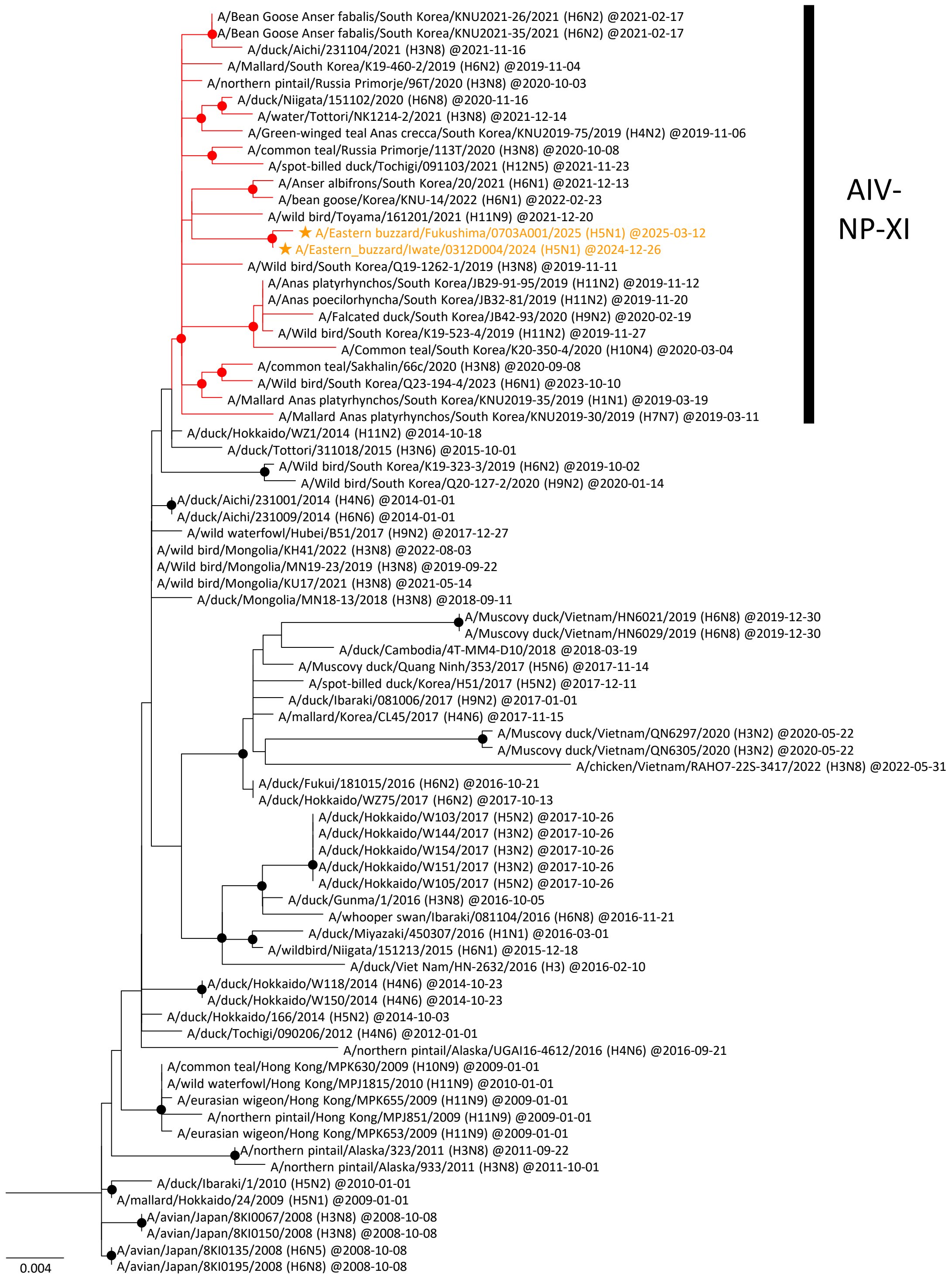

Fig. S1p. Partial nucleoprotein (NP) gene phylogenic tree showing the AIV-NP-XI cluster.

Isolates marked with a yellow star (★) represent isolates derived from wild birds collected in Japan during the 2024/25 season. Filled circles (●) at nodes indicate Shimodaira–Hasegawa approximate likelihood ratio test (SH-aLRT)/bootstrap support values of ≥80/95.

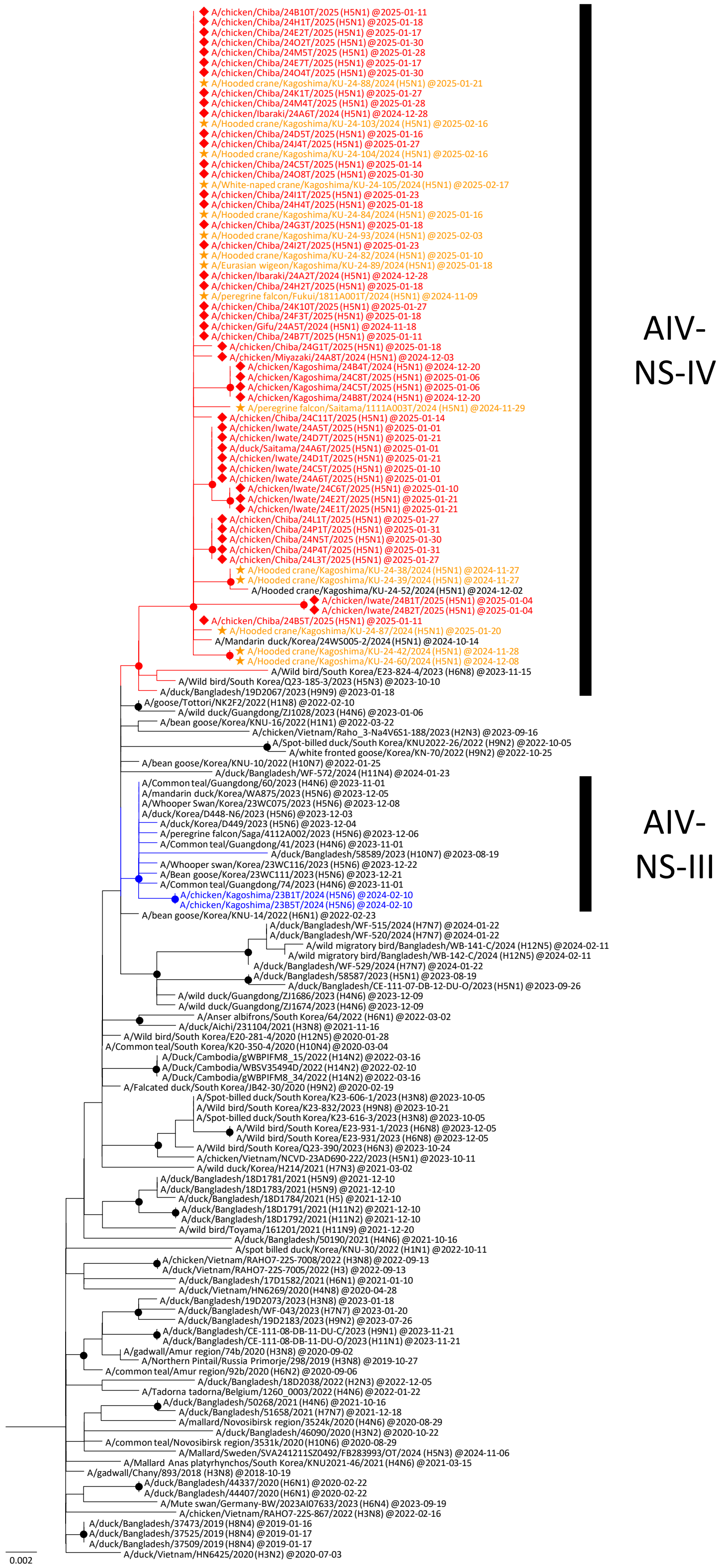

Fig. S1q. Partial nonstructural protein (NS) gene phylogenic tree showing the AIV-NS-III and IV cluster.

Isolates marked with a red diamond (◆) represent isolates derived from Japanese poultry and analyzed at the National Institute of Animal Health (NIAH) in the 2024/25 season, and those marked with a yellow star (★) represent isolates derived from wild birds collected in Japan during the same season. Isolates colored in blue represent isolates from Japanese poultry analyzed at the NIAH in the 2023/24 season. Filled circles (●) at nodes indicate Shimodaira–Hasegawa approximate likelihood ratio test (SH-aLRT)/bootstrap support values of ≥80/95.

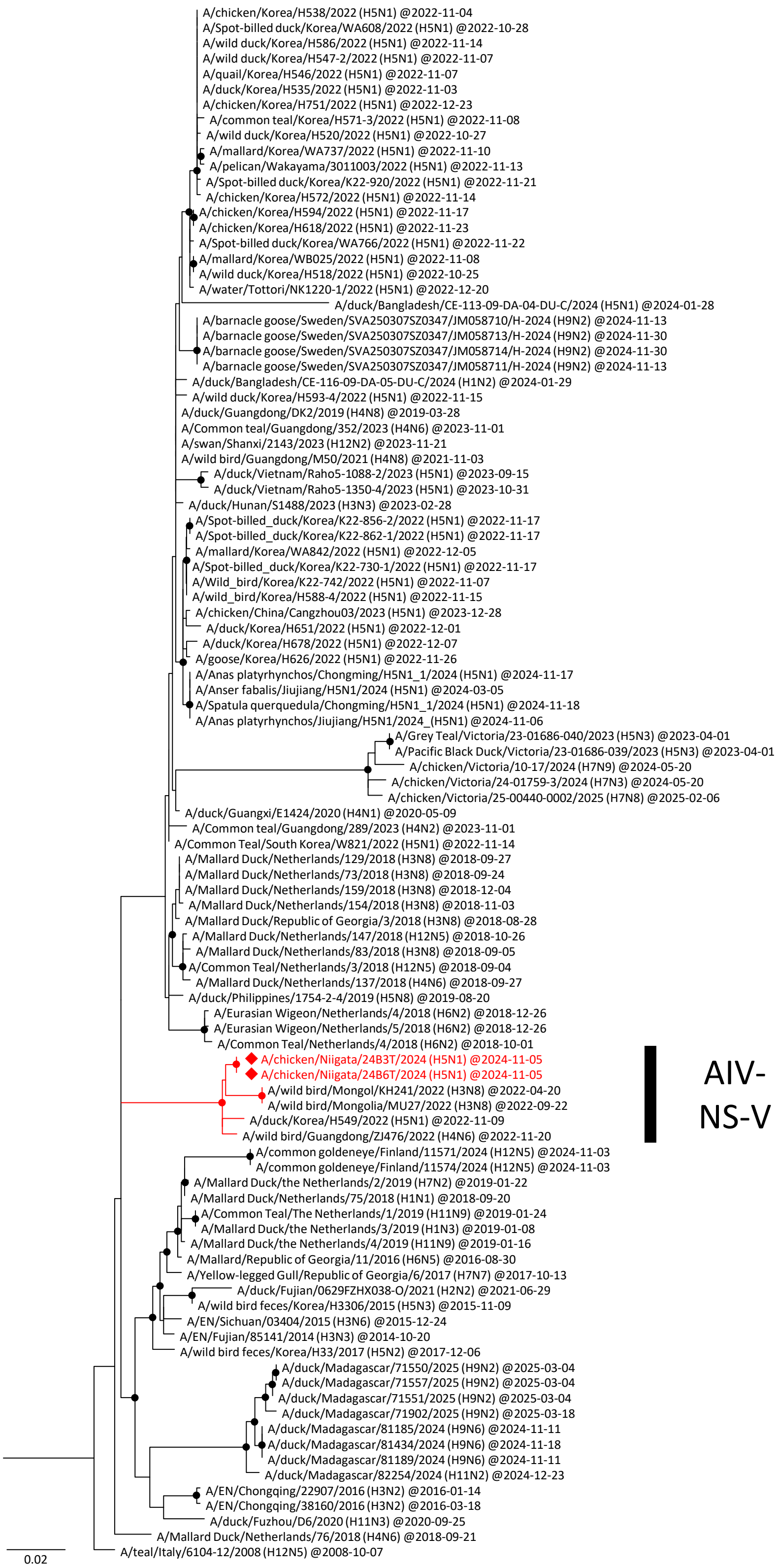

Fig. S1r. Partial nonstructural protein (NS) gene phylogenic tree showing the AIV-NS-V cluster.

Isolates marked with a red diamond (◆) represent isolates derived from Japanese poultry and analyzed at the National Institute of Animal Health (NIAH) in the 2024/25 season. Filled circles (●) at nodes indicate Shimodaira–Hasegawa approximate likelihood ratio test (SH-aLRT)/bootstrap support values of ≥80/95.

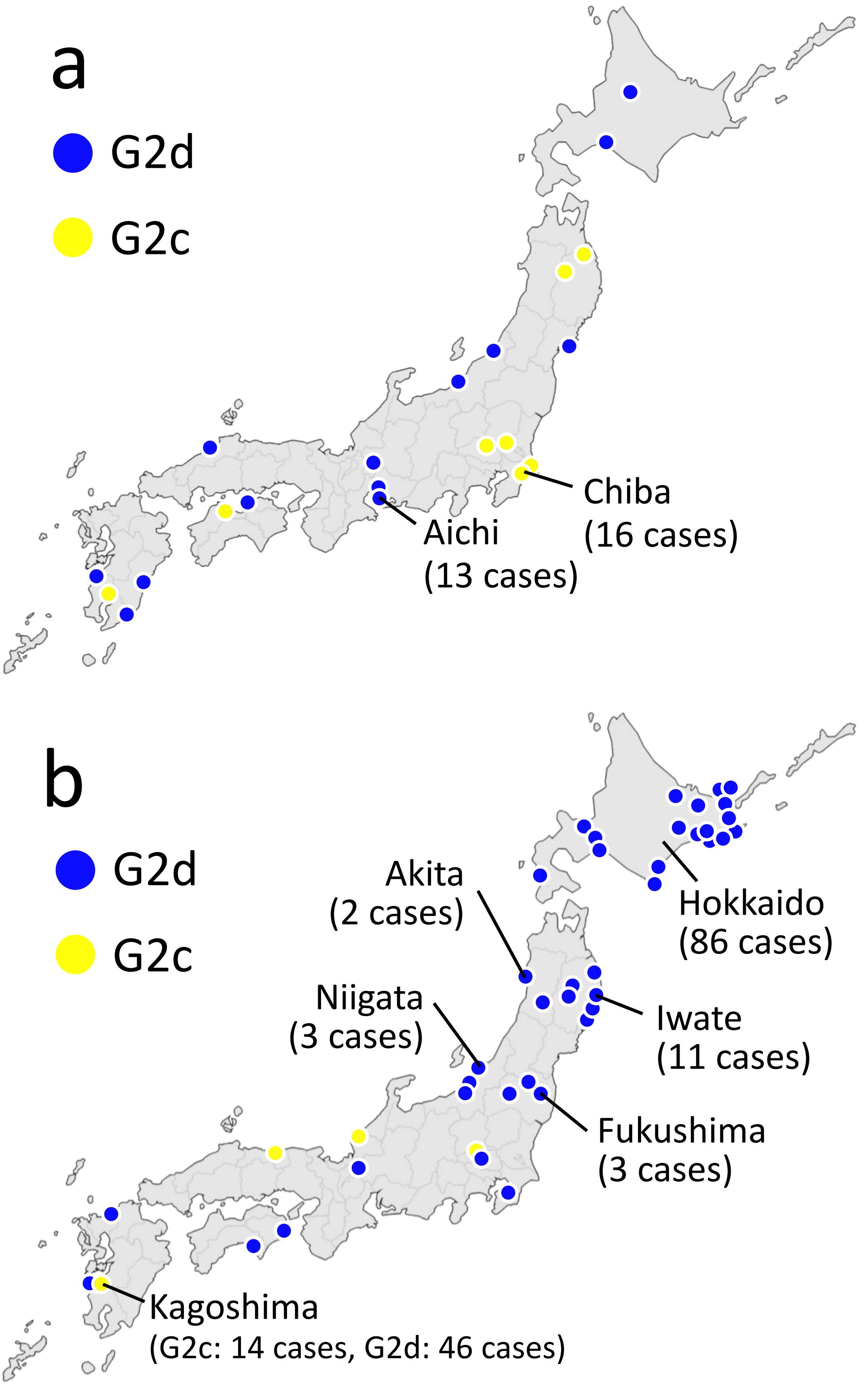

Fig. S2. Geographic distribution of cities in Japan with confirmed high pathogenicity avian influenza (HPAI) cases during the 2023–2024 season. Cases detected in: (a) poultry; (b) wild birds.

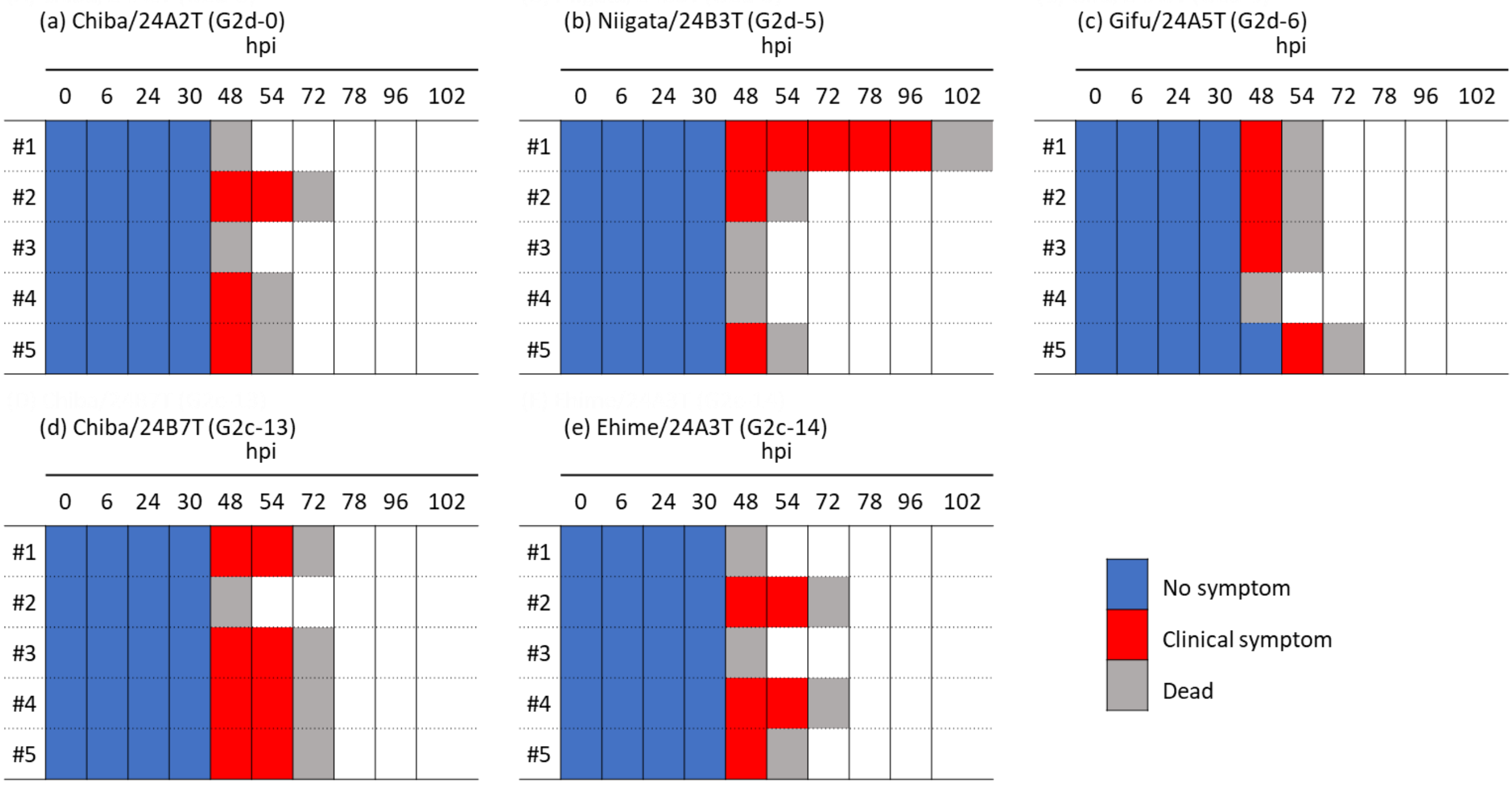

Fig. S3. Detailed history of five chickens inoculated with 10^6 50% egg infectious dose (EID_{50}) of representative virus strains.

Blue boxes, no clinical sign; red boxes, clinical signs; gray boxes, observed time of death; hpi, hours post-inoculation.

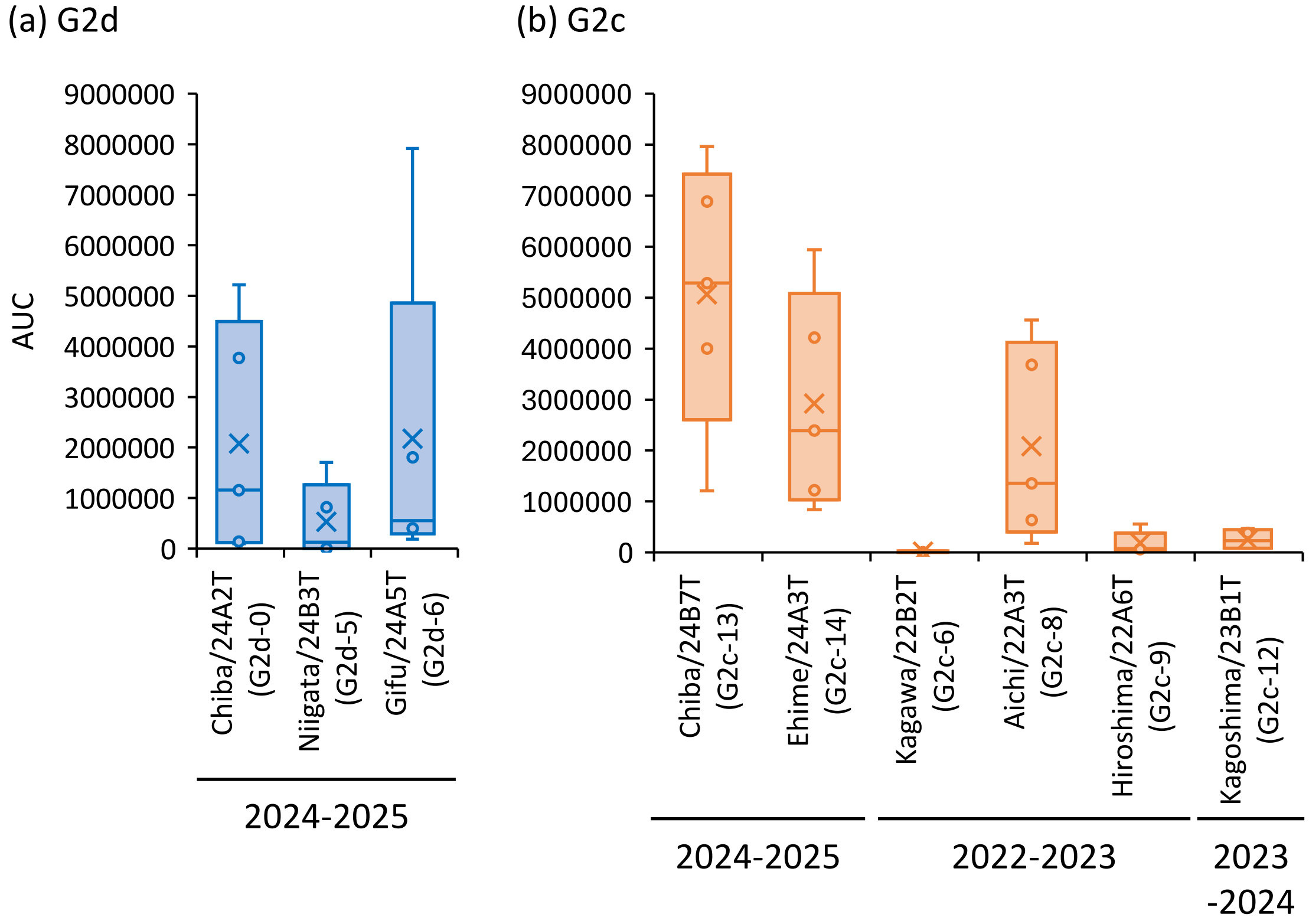

Fig. S4. Comparison of area under the curve (AUC) of viral titers in the 10^6 inoculation group.

Box plots indicate the AUC of viral titers from tracheal and cloacal swabs of chickens infected with: (a) G2d and (b) G2c viruses. Data for Kagoshima/23B1T were retrieved from a previous study (Nishiura et al., 2024).
