# Supplementary material for: High pathogenicity avian influenza A (H5N1) viruses isolated from poultry and wild birds in Japan during the 2024–2025 season
Source: Microbiol Spectr. 2026 Jul 14;14(8):e00293-26. doi: 10.1128/spectrum.00293-26 (PMC13435830; doi:10.1128/spectrum.00293-26)
Supplement: Supplementary file 2 [file spectrum.00293-26-s0001.docx]

**Supplemental tables**

**Table S1. Detailed information for each isolate collected from poultry during the 2024–2025 season**

| Number | Prefecture | Date | Genotype | Representative strain | Isolate ID |
| --- | --- | --- | --- | --- | --- |
| 1 | Hokkaido | October 17, 2024 | G2d-0 | A/chicken/Hokkaido/24A1T/2024 | EPI_ISL_19655349 |
|  |  |  |  | A/chicken/Hokkaido/24A2T/2024 | EPI_ISL_19655350 |
| 2 | Chiba | October 23, 2024 | G2d-0 | A/chicken/Chiba/24A2T/2024 | EPI_ISL_19655351 |
|  |  |  |  | A/chicken/Chiba/24A4T/2024 | EPI_ISL_19655352 |
| 3 | Niigata | October 26, 2024 | G2d-0 | A/chicken/Niigata/24A3T/2024 | EPI_ISL_19655353 |
|  |  |  |  | A/chicken/Niigata/24A8T/2024 | EPI_ISL_19655354 |
| 4 | Shimane | October 31, 2024 | G2d-0 | A/chicken/Simane/24A4T/2024 | EPI_ISL_19655418 |
|  |  |  |  | A/chicken/Simane/24A4C/2024 | EPI_ISL_19655420 |
| 5 | Niigata | November 6, 2024 | G2d-5 | A/chicken/Niigata/24B3T/2024 | EPI_ISL_19655422 |
|  |  |  |  | A/chicken/Niigata/24B6T/2024 | EPI_ISL_19655424 |
| 6 | Kagawa | November 7, 2024 | G2d-0 | A/chicken/Kagawa/24A4T/2024 | EPI_ISL_19655426 |
|  |  |  |  | A/chicken/Kagawa/24A7T/2024 | EPI_ISL_19655429 |
| 7 | Miyagi | November 10, 2024 | G2d-0 | A/chicken/Miyagi/24A5T/2024 | EPI_ISL_19655431 |
|  |  |  |  | A/chicken/Miyagi/24A8T/2024 | EPI_ISL_19655434 |
| 8 | Hokkaido | November 12, 2024 | G2d-0 | A/chicken/Hokkaido/24B1T/2024 | EPI_ISL_19655436 |
|  |  |  |  | A/chicken/Hokkaido/24B10T/2024 | EPI_ISL_19655466 |
| 9 | Gifu | November 19, 2024 | G2d-6 | A/chicken/Gifu/24A5T/2024 | EPI_ISL_19655606 |
| 10 | Kagoshima | November 20, 2024 | G2d-0 | A/chicken/Kagoshima/24A2T/2024 | EPI_ISL_19655645 |
|  |  |  |  | A/chicken/Kagoshima/24A7T/2024 | EPI_ISL_19655696 |
| 11 | Saitama | November 25, 2024 | G2c-13 | A/duck/Saitama/24A6T/2024 | EPI_ISL_19655829 |
| 12 | Miyazaki | December 3, 2024 | G2d-6 | A/chicken/Miyazaki/24A8T/2024 | EPI_ISL_20158068 |
| 13 | Ehime | December 10, 2024 | G2c-14 | A/chicken/Ehime/24A3T/2024 | EPI_ISL_20084482 |
|  |  |  |  | A/chicken/Ehime/24A6T/2024 | EPI_ISL_20158069 |
| 14 | Ehime | December 19, 2024 | G2c-14 | A/chicken/Ehime/24B12T/2024 | EPI_ISL_20158070 |
|  |  |  |  | A/chicken/Ehime/24B16T/2024 | EPI_ISL_20158071 |
| 15 | Kagoshima | December 20, 2024 | G2c-13 | A/chicken/Kagoshima/24B4T/2024 | EPI_ISL_20158072 |
|  |  |  |  | A/chicken/Kagoshima/24B8T/2024 | EPI_ISL_20158073 |
| 16 | Ibaraki | December 29, 2024 | G2c-13 | A/chicken/Ibaraki/24A2T/2024 | EPI_ISL_20158074 |
|  |  |  |  | A/chicken/Ibaraki/24A6T/2024 | EPI_ISL_20158075 |
| 17 | Aichi | January 2, 2025 | G2d-0 | A/chicken/Aichi/24A3T/2025 | EPI_ISL_20158076 |
|  |  |  |  | A/chicken/Aichi/24A6T/2025 | EPI_ISL_20158077 |
| 18 | Iwate | January 2, 2025 | G2c-13 | A/chicken/Iwate/24A5T/2025 | EPI_ISL_20158078 |
|  |  |  |  | A/chicken/Iwate/24A6T/2025 | EPI_ISL_20158079 |
| 19 | Iwate | January 5, 2025 | G2c-13 | A/chicken/Iwate/24B1T/2025 | EPI_ISL_20158080 |
|  |  |  |  | A/chicken/Iwate/24B2T/2025 | EPI_ISL_20158081 |
| 20 | Aichi | January 6, 2025 | G2d-0 | A/chicken/Aichi/24B7T/2025 | EPI_ISL_20158082 |
|  |  |  |  | A/chicken/Aichi/24B8T/2025 | EPI_ISL_20158083 |
| 21 | Kagoshima | January 7, 2025 | G2c-13 | A/chicken/Kagoshima/24C5T/2025 | EPI_ISL_20158084 |
|  |  |  |  | A/chicken/Kagoshima/24C8T/2025 | EPI_ISL_20158085 |
| 22 | Aichi | January 9, 2025 | G2d-0 | A/chicken/Aichi/24C2T/2025 | EPI_ISL_20158086 |
|  |  |  |  | A/chicken/Aichi/24C8T/2025 | EPI_ISL_20158087 |
| 23 | Aichi | January 10, 2025 | G2d-0 | A/chicken/Aichi/24D4T/2025 | EPI_ISL_20158088 |
|  |  |  |  | A/chicken/Aichi/24D8T/2025 | EPI_ISL_20158089 |
| 24 | Aichi | January 10, 2025 | G2d-0 | A/chicken/Aichi/24E2T/2025 | EPI_ISL_20158090 |
|  |  |  |  | A/chicken/Aichi/24E10T/2025 | EPI_ISL_20158091 |
| 25 | Miyazaki | January 11, 2025 | G2d-0 | A/chicken/Miyazaki/24B5T/2025 | EPI_ISL_20158098 |
|  |  |  |  | A/chicken/Miyazaki/24B6T/2025 | EPI_ISL_20158103 |
| 26 | Iwate | January 11, 2025 | G2c-13 | A/chicken/Iwate/24C5T/2025 | EPI_ISL_20158104 |
|  |  |  |  | A/chicken/Iwate/24C6T/2025 | EPI_ISL_20158105 |
| 27 | Aichi | January 11, 2025 | G2d-0 | A/chicken/Aichi/24F1T/2025 | EPI_ISL_20158106 |
|  |  |  |  | A/chicken/Aichi/24F8T/2025 | EPI_ISL_20158107 |
| 28 | Chiba | January 12, 2025 | G2c-13 | A/chicken/Chiba/24B5T/2025 | EPI_ISL_20084480 |
|  |  |  |  | A/chicken/Chiba/24B7T/2025 | EPI_ISL_20158108 |
|  |  |  |  | A/chicken/Chiba/24B10T/2025 | EPI_ISL_20158109 |
| 29 | Chiba | January 15, 2025 | G2c-13 | A/chicken/Chiba/24C5T/2025 | EPI_ISL_20158110 |
|  |  |  |  | A/chicken/Chiba/24C11T/2025 | EPI_ISL_20158111 |
| 30 | Aichi | January 16, 2025 | G2d-0 | A/chicken/Aichi/24G3T/2025 | EPI_ISL_20158115 |
|  |  |  |  | A/chicken/Aichi/24G4T/2025 | EPI_ISL_20158116 |
| 31 | Chiba | January 16, 2025 | G2c-13 | A/chicken/Chiba/24D5T/2025 | EPI_ISL_20158117 |
| 32 | Chiba | January 18, 2025 | G2c-13 | A/chicken/Chiba/24E2T/2025 | EPI_ISL_20158118 |
|  |  |  |  | A/chicken/Chiba/24E7T/2025 | EPI_ISL_20158119 |
| 33 | Aichi | January 19, 2025 | G2d-0 | A/chicken/Aichi/24H1T/2025 | EPI_ISL_20158120 |
|  |  |  |  | A/chicken/Aichi/24H2T/2025 | EPI_ISL_20158121 |
| 34 | Aichi | January 19, 2025 | G2d-0 | A/chicken/Aichi/24I4T/2025 | EPI_ISL_20158122 |
|  |  |  |  | A/chicken/Aichi/24I8T/2025 | EPI_ISL_20158126 |
| 35 | Aichi | January 19, 2025 | G2d-0 | A/chicken/Aichi/24J9T/2025 | EPI_ISL_20158147 |
|  |  |  |  | A/chicken/Aichi/24J10T/2025 | EPI_ISL_20158148 |
| 36 | Aichi | January 19, 2025 | G2d-0 | A/quail/Aichi/24K3T/2025 | EPI_ISL_20158149 |
|  |  |  |  | A/quail/Aichi/24K8T/2025 | EPI_ISL_20158150 |
| 37 | Chiba | January 19, 2025 | G2c-13 | A/chicken/Chiba/24F3T/2025 | EPI_ISL_20158151 |
| 38 | Chiba | January 19, 2025 | G2c-13 | A/chicken/Chiba/24G1T/2025 | EPI_ISL_20158152 |
|  |  |  |  | A/chicken/Chiba/24G3T/2025 | EPI_ISL_20158153 |
| 39 | Chiba | January 19, 2025 | G2c-13 | A/chicken/Chiba/24H1T/2025 | EPI_ISL_20158154 |
|  |  |  |  | A/chicken/Chiba/24H2T/2025 | EPI_ISL_20158155 |
|  |  |  |  | A/chicken/Chiba/24H4T/2025 | EPI_ISL_20158156 |
| 40 | Aichi | January 21, 2025 | G2d-0 | A/chicken/Aichi/24L1T/2025 | EPI_ISL_20158157 |
| 41 | Iwate | January 22, 2025 | G2c-13 | A/chicken/Iwate/24D1T/2025 | EPI_ISL_20158158 |
|  |  |  |  | A/chicken/Iwate/24D7T/2025 | EPI_ISL_20158159 |
| 42 | Iwate | January 22, 2025 | G2c-13 | A/chicken/Iwate/24E1T/2025 | EPI_ISL_20158160 |
|  |  |  |  | A/chicken/Iwate/24E2T/2025 | EPI_ISL_20158161 |
| 43 | Chiba | January 24, 2025 | G2c-13 | A/chicken/Chiba/24I1T/2025 | EPI_ISL_20158162 |
|  |  |  |  | A/chicken/Chiba/24I2T/2025 | EPI_ISL_20158163 |
| 44 | Chiba | January 28, 2025 | G2c-13 | A/chicken/Chiba/24J4T/2025 | EPI_ISL_20158164 |
| 45 | Chiba | January 28, 2025 | G2c-13 | A/chicken/Chiba/24K10T/2025 | EPI_ISL_20158165 |
|  |  |  |  | A/chicken/Chiba/24K1T/2025 | EPI_ISL_20158166 |
| 46 | Chiba | January 28, 2025 | G2c-13 | A/chicken/Chiba/24L1T/2025 | EPI_ISL_20158167 |
|  |  |  |  | A/chicken/Chiba/24L3T/2025 | EPI_ISL_20158168 |
| 47 | Chiba | January 29, 2025 | G2c-13 | A/chicken/Chiba/24M4T/2025 | EPI_ISL_20158169 |
|  |  |  |  | A/chicken/Chiba/24M5T/2025 | EPI_ISL_20158170 |
| 48 | Aichi | January 31, 2025 | G2d-0 | A/chicken/Aichi/24M5T/2025 | EPI_ISL_20158171 |
|  |  |  |  | A/chicken/Aichi/24M8T/2025 | EPI_ISL_20158172 |
| 49 | Chiba | January 31, 2025 | G2c-13 | A/chicken/Chiba/24N5T/2025 | EPI_ISL_20158173 |
| 50 | Chiba | January 31, 2025 | G2c-13 | A/chicken/Chiba/24O2T/2025 | EPI_ISL_20158174 |
|  |  |  |  | A/chicken/Chiba/24O4T/2025 | EPI_ISL_20158175 |
|  |  |  |  | A/chicken/Chiba/24O8T/2025 | EPI_ISL_20158176 |
| 51 | Chiba | February 1, 2025 | G2c-13 | A/chicken/Chiba/24P1T/2025 | EPI_ISL_20158177 |
|  |  |  |  | A/chicken/Chiba/24P4T/2025 | EPI_ISL_20158178 |

**Table S2. Detailed information for each isolate collected from wild birds during the 2024–2025 season**

| Number | Prefecture | Date | Wild bird species | Genotype | Representative strain | Isolate ID | Originating institute |
| --- | --- | --- | --- | --- | --- | --- | --- |
| 1 | Hokkaido | September 30, 2024 | peregrine falcon | G2d-0 | A/peregrine falcon/Hokkaido/4809Z011T/2024 | EPI_ISL_19498313 | National Institute of Animal Health |
| 2 | Hokkaido | October 8, 2024 | Eurasian wigeon | G2d-0 | A/Eurasian wigeon/Hokkaido/Q76/2024 | EPI_ISL_19476809 | Hokkaido University |
| 3 | Hokkaido | October 21, 2024 | white-tailed eagle | G2d-0 | A/white-tailed eagle/Hokkaido/20241016002/2024 | EPI_ISL_19504792 | Hokkaido University |
| 4 | Niigata | October 16, 2024 | northern goshawk | G2d-0 | A/northern goshawk/Niigata/1510C001T/2024 | EPI_ISL_20111727 | National Institute of Animal Health |
| 5 | Akita | October 16, 2024 | common teal | G2d-0 | A/common teal/Akita/0510D002T/2024 | EPI_ISL_20111729 | National Institute of Animal Health |
| 6 | Hokkaido | October 22, 2024 | red-crowned crane | G2d-0 | A/red-crowned crane/Hokkaido/1022-001/2024 | EPI_ISL_20095289 | Tottori University |
| 7 | Hokkaido | October 24, 2024 | whooper swan | G2d-0 | A/whooper swan/Hokkaido/0110M131/2024 | EPI_ISL_20093504 | Tottori University |
| 8 | Tokushima | October 24, 2024 | Eurasian wigeon | G2d-0 | A/Eurasian_wigeon/Tokushima/3610A002T/2024 | EPI_ISL_20111732 | National Institute of Animal Health |
| 9 | Shiga | October 25, 2024 | peregrine falcon | G2d-0 | A/peregrine falcon/Shiga/2510T002T/2024 | EPI_ISL_20111730 | National Institute of Animal Health |
| 10 | Hokkaido | October 25, 2024 | whooper swan | G2d-0 | A/whooper swan/Hokkaido/0104P119/2024 | EPI_ISL_19700696 | Kagoshima University |
| 11 | Hokkaido | October 30, 2024 | whooper swan | G2d-0 | A/whooper swan/Hokkaido/0104P120/2024 | EPI_ISL_19574220 | Hokkaido University |
| 12 | Hokkaido | October 30, 2024 | whooper swan | G2d-0 | A/whooper swan/Hokkaido/0110N164/2024 | EPI_ISL_19574223 | Hokkaido University |
| 13 | Hokkaido | October 31, 2024 | whooper swan | G2d-0 | A/whooper swan/Hokkaido/0110N165/2024 | EPI_ISL_19574224 | Hokkaido University |
| 14 | Akita | October 31, 2024 | whooper swan | G2d-0 | A/whooper_swan/Akita/0510G004/2024 | EPI_ISL_20095299 | Tottori University |
| 15 | Hokkaido | October 31, 2024 | whooper swan | G2d-0 | A/whooper swan/Hokkaido/0110M133/2024 | EPI_ISL_19850332 | Kagoshima University |
| 16 | Hokkaido | October 31, 2024 | peregrine falcon | G2d-0 | A/peregrine_falcon/Hokkaido/20241031001/2024 | EPI_ISL_19700697 | Kagoshima University |
| 17 | Hokkaido | November 1, 2024 | red-crowned crane | G2d-0 | A/red-crowned crane/Hokkaido/1101-001/2024 | EPI_ISL_20095303 | Tottori University |
| 18 | Niigata | November 1, 2024 | northern goshawk | G2d-0 | A/northern_goshawk/Niigata/1511C001/2024 | EPI_ISL_20095287 | Tottori University |
| 19 | Fukuoka | November 1, 2024 | Eurasian wigeon | G2d-0 | A/Eurasian wigeon/Fukuoka/4011A001T/2024 | EPI_ISL_20111766 | National Institute of Animal Health |
| 20 | Niigata | November 3, 2024 | tufted duck | G2d-0 | A/tufted duck/Niigata/5111001/2024 | EPI_ISL_19574221 | Hokkaido University |
| 21 | Tottori | November 7, 2024 | duck | G2c-13 | A/duck/Tottori/NK11F5-8/2024 | EPI_ISL_20096712 | Tottori University |
| 22 | Fukui | November 9, 2024 | peregrine falcon | G2c-14 | A/peregrine falcon/Fukui/1811A001T/2024 | EPI_ISL_20111740 | National Institute of Animal Health |
| 23 | Hokkaido | November 10, 2024 | large-billed crow | G2d-0 | A/large-billed crow/Hokkaido/0111B127/2024 | EPI_ISL_19574222 | Hokkaido University |
| 24 | Kumamoto | November 12, 2024 | Eurasian wigeon | G2d-0 | A/Eurasian wigeon/Kumamoto/4311J002T/2024 | EPI_ISL_20111781 | National Institute of Animal Health |
| 25 | Kagoshima | November 16, 2024 | hooded crane | G2d-0 | A/hooded crane/Kagoshima/KU-24-1/2024 | EPI_ISL_19624026 | Kagoshima University |
| 26 | Kagoshima | November 17, 2024 | hooded crane | G2d-0 | A/hooded crane/Kagoshima/KU-24-2/2024 | EPI_ISL_19624025 | Kagoshima University |
| 27 | Kagoshima | November 18, 2024 | hooded crane | G2d-0 | A/hooded crane/Kagoshima/KU-24-3/2024 | EPI_ISL_19645340 | Kagoshima University |
| 28 | Kagoshima | November 18, 2024 | hooded crane | G2d-0 | A/hooded crane/Kagoshima/KU-24-6/2024 | EPI_ISL_19645362 | Kagoshima University |
| 29 | Kagoshima | November 18, 2024 | hooded crane | G2d-0 | A/hooded crane/Kagoshima/KU-24-7/2024 | EPI_ISL_19645363 | Kagoshima University |
| 30 | Kagoshima | November 18, 2024 | Eurasian wigeon | G2d-0 | A/Eurasian_wigeon/Kagoshima/KU-24-4/2024 | EPI_ISL_19645341 | Kagoshima University |
| 31 | Kagoshima | November 19, 2024 | hooded crane | G2d-0 | A/hooded crane/Kagoshima/KU-24-8/2024 | EPI_ISL_19645364 | Kagoshima University |
| 32 | Kagoshima | November 19, 2024 | hooded crane | G2d-0 | A/hooded crane/Kagoshima/KU-24-9/2024 | EPI_ISL_19700683 | Kagoshima University |
| 33 | Kagoshima | November 19, 2024 | hooded crane | G2d-0 | A/hooded crane/Kagoshima/KU-24-10/2024 | EPI_ISL_19707515 | Kagoshima University |
| 34 | Kagoshima | November 19, 2024 | hooded crane | G2d-0 | A/hooded crane/Kagoshima/KU-24-11/2024 | EPI_ISL_19700684 | Kagoshima University |
| 35 | Kagoshima | November 19, 2024 | hooded crane | G2d-0 | A/hooded crane/Kagoshima/KU-24-26/2024 | EPI_ISL_19700691 | Kagoshima University |
| 36 | Kagoshima | November 20, 2024 | hooded crane | G2d-0 | A/hooded crane/Kagoshima/KU-24-13/2024 | EPI_ISL_19700406 | Kagoshima University |
| 37 | Kagoshima | November 20, 2024 | hooded crane | G2d-0 | A/hooded crane/Kagoshima/KU-24-14/2024 | EPI_ISL_19700407 | Kagoshima University |
| 38 | Hokkaido | November 20, 2024 | carrion crow | G2d-0 | A/carrion crow/Hokkaido/0111Q112/2024 | EPI_ISL_19666681 | Hokkaido University |
| 39 | Kagoshima | November 21, 2024 | hooded crane | G2d-0 | A/hooded crane/Kagoshima/KU-24-12/2024 | EPI_ISL_19645342 | Kagoshima University |
| 40 | Kagoshima | November 21, 2024 | hooded crane | G2d-0 | A/hooded crane/Kagoshima/KU-24-15/2024 | EPI_ISL_19645346 | Kagoshima University |
| 41 | Kagoshima | November 21, 2024 | hooded crane | G2d-0 | A/hooded crane/Kagoshima/KU-24-17/2024 | EPI_ISL_19645348 | Kagoshima University |
| 42 | Kagoshima | November 21, 2024 | hooded crane | G2d-0 | A/hooded crane/Kagoshima/KU-24-19/2024 | EPI_ISL_19700408 | Kagoshima University |
| 43 | Kagoshima | November 21, 2024 | hooded crane | G2d-0 | A/hooded crane/Kagoshima/KU-24-20/2024 | EPI_ISL_19700409 | Kagoshima University |
| 44 | Kagoshima | November 21, 2024 | hooded crane | G2d-0 | A/hooded crane/Kagoshima/KU-24-21/2024 | EPI_ISL_19700410 | Kagoshima University |
| 45 | Kagoshima | November 21, 2024 | hooded crane | G2d-0 | A/hooded crane/Kagoshima/KU-24-22/2024 | EPI_ISL_19707505 | Kagoshima University |
| 46 | Kagoshima | November 21, 2024 | white-naped crane | G2d-0 | A/white-naped crane/Kagoshima/KU-24-16/2024 | EPI_ISL_19645347 | Kagoshima University |
| 47 | Hokkaido | November 21, 2024 | carrion crow | G2d-0 | A/carrion crow/Hokkaido/0111Q112/2024 | EPI_ISL_19666681 | Hokkaido University |
| 48 | Kagoshima | November 22, 2024 | hooded crane | G2d-0 | A/hooded crane/Kagoshima/KU-24-18/2024 | EPI_ISL_19645349 | Kagoshima University |
| 49 | Kagoshima | November 22, 2024 | hooded crane | G2d-0 | A/hooded crane/Kagoshima/KU-24-23/2024 | EPI_ISL_19700685 | Kagoshima University |
| 50 | Kagoshima | November 22, 2024 | hooded crane | G2d-7 | A/hooded crane/Kagoshima/KU-24-24/2024 | EPI_ISL_19700686 | Kagoshima University |
| 51 | Kagoshima | November 22, 2024 | hooded crane | G2d-0 | A/hooded crane/Kagoshima/KU-24-25/2024 | EPI_ISL_19700687 | Kagoshima University |
| 52 | Hokkaido | November 22, 2024 | whooper swan | G2d-0 | A/whooper swan/Hokkaido/20241122001/2024 | EPI_ISL_19666579 | Hokkaido University |
| 53 | Kagoshima | November 23, 2024 | hooded crane | G2d-0 | A/hooded crane/Kagoshima/KU-24-27/2024 | EPI_ISL_19707516 | Kagoshima University |
| 54 | Kagoshima | November 23, 2024 | hooded crane | G2d-0 | A/hooded crane/Kagoshima/KU-24-28/2024 | EPI_ISL_19700692 | Kagoshima University |
| 55 | Kagoshima | November 24, 2024 | hooded crane | G2d-0 | A/hooded crane/Kagoshima/KU-24-29/2024 | EPI_ISL_19700693 | Kagoshima University |
| 56 | Kagoshima | November 25, 2024 | hooded crane | G2d-0 | A/hooded crane/Kagoshima/KU-24-30/2024 | EPI_ISL_20051053 | Kagoshima University |
| 57 | Kagoshima | November 25, 2024 | hooded crane | G2d-0 | A/hooded crane/Kagoshima/KU-24-31/2024 | EPI_ISL_19700694 | Kagoshima University |
| 58 | Kagoshima | November 25, 2024 | hooded crane | G2d-0 | A/hooded crane/Kagoshima/KU-24-32/2024 | EPI_ISL_19707506 | Kagoshima University |
| 59 | Hokkaido | November 27, 2024 | carrion crow | G2d-0 | A/carrion_crow/Hokkaido/0111Q114/2024 | EPI_ISL_20095304 | Tottori University |
| 60 | Kagoshima | November 27, 2024 | hooded crane | G2c-13 | A/hooded crane/Kagoshima/KU-24-38/2024 | EPI_ISL_19707507 | Kagoshima University |
| 61 | Kagoshima | November 27, 2024 | hooded crane | G2c-13 | A/hooded crane/Kagoshima/KU-24-39/2024 | EPI_ISL_19700695 | Kagoshima University |
| 62 | Kagoshima | November 28, 2024 | hooded crane | G2d-0 | A/hooded crane/Kagoshima/KU-24-40/2024 | EPI_ISL_19707508 | Kagoshima University |
| 63 | Kagoshima | November 28, 2024 | hooded crane | G2d-0 | A/hooded crane/Kagoshima/KU-24-41/2024 | EPI_ISL_19707509 | Kagoshima University |
| 64 | Kagoshima | November 28, 2024 | hooded crane | G2c-15 | A/hooded crane/Kagoshima/KU-24-42/2024 | EPI_ISL_19707510 | Kagoshima University |
| 65 | Kagoshima | November 29, 2024 | hooded crane | G2d-0 | A/hooded crane/Kagoshima/KU-24-43/2024 | EPI_ISL_19707511 | Kagoshima University |
| 66 | Kagoshima | November 29, 2024 | hooded crane | G2d-0 | A/hooded crane/Kagoshima/KU-24-46/2024 | EPI_ISL_19707517 | Kagoshima University |
| 67 | Kagoshima | November 29, 2024 | white-naped crane | G2d-0 | A/white-naped crane/Kagoshima/KU-24-44/2024 | EPI_ISL_19707512 | Kagoshima University |
| 68 | Saitama | November 29, 2024 | peregrine falcon | G2c-16 | A/peregrine falcon/Saitama/1111A003T/2024 | EPI_ISL_20111800 | National Institute of Animal Health |
| 69 | Kagoshima | November 30, 2024 | hooded crane | G2d-8 | A/hooded crane/Kagoshima/KU-24-47/2024 | EPI_ISL_19707518 | Kagoshima University |
| 70 | Kagoshima | November 30, 2024 | hooded crane | G2d-0 | A/hooded crane/Kagoshima/KU-24-48/2024 | EPI_ISL_19707519 | Kagoshima University |
| 71 | Hokkaido | December 1, 2024 | large-billed crow | G2d-0 | A/large-billed crow/Hokkaido/0112M136/2024 | EPI_ISL_19776461 | Kagoshima University |
| 72 | Hokkaido | December 2, 2024 | large-billed crow | G2d-0 | A/large-billed crow/Hokkaido/0112F071/2024 | EPI_ISL_19850333 | Kagoshima University |
| 73 | Hokkaido | December 2, 2024 | large-billed crow | G2d-0 | A/large-billed crow/Hokkaido/0112F072/2024 | EPI_ISL_19776463 | Kagoshima University |
| 74 | Hokkaido | December 2, 2024 | large-billed crow | G2d-0 | A/large-billed crow/Hokkaido/0112F070/2024 | EPI_ISL_19776462 | Kagoshima University |
| 75 | Kagoshima | December 2, 2024 | white-naped crane | G2d-0 | A/white-naped crane/Kagoshima/KU-24-53/2024 | EPI_ISL_19707521 | Kagoshima University |
| 76 | Kagoshima | December 2, 2024 | hooded crane | G2c-13 | A/hooded crane/Kagoshima/KU-24-52/2024 | EPI_ISL_19707520 | Kagoshima University |
| 77 | Hokkaido | December 2, 2024 | large-billed crow | G2d-0 | A/large-billed crow/Hokkaido/0112F073/2024 | EPI_ISL_19666680 | Hokkaido University |
| 78 | Kagoshima | December 3, 2024 | hooded crane | G2d-0 | A/hooded crane/Kagoshima/KU-24-54/2024 | EPI_ISL_19707522 | Kagoshima University |
| 79 | Kagoshima | December 3, 2024 | hooded crane | G2d-0 | A/hooded crane/Kagoshima/KU-24-56/2024 | EPI_ISL_19769499 | Kagoshima University |
| 80 | Hokkaido | December 4, 2024 | large-billed crow | G2d-0 | A/large-billed crow/Hokkaido/0112F074/2024 | EPI_ISL_19666679 | Hokkaido University |
| 81 | Kagoshima | December 6, 2024 | hooded crane | G2d-0 | A/hooded crane/Kagoshima/KU-24-58/2024 | EPI_ISL_19769500 | Kagoshima University |
| 82 | Kagoshima | December 6, 2024 | hooded crane | G2d-0 | A/hooded crane/Kagoshima/KU-24-62/2024 | EPI_ISL_19769502 | Kagoshima University |
| 83 | Kagoshima | December 8, 2024 | hooded crane | G2c-15 | A/hooded crane/Kagoshima/KU-24-60/2024 | EPI_ISL_19769501 | Kagoshima University |
| 84 | Kagoshima | December 9, 2024 | hooded crane | G2d-0 | A/hooded crane/Kagoshima/KU-24-63/2024 | EPI_ISL_19769503 | Kagoshima University |
| 85 | Hokkaido | December 9, 2024 | large-billed crow | G2d-0 | A/large-billed crow/Hokkaido/0112N167/2024 | EPI_ISL_19666667 | Hokkaido University |
| 86 | Chiba | December 10, 2024 | duck | G2d-0 | A/duck/Chiba/24e10Fwb/2024 | EPI_ISL_20111837 | National Institute of Animal Health |
| 87 | Iwate | December 11, 2024 | whooper swan | G2d-0 | A/whooper_swan/Iwate/0312K004/2024 | EPI_ISL_20095323 | Tottori University |
| 88 | Hokkaido | December 11, 2024 | large-billed crow | G2d-0 | A/large-billed_crow/Hokkaido/0112F075/2024 | EPI_ISL_20095324 | Tottori University |
| 89 | Hokkaido | December 13, 2024 | large-billed crow | G2d-0 | A/large-billed crow/Hokkaido/B169/2024 | EPI_ISL_19666665 | Hokkaido University |
| 90 | Hokkaido | December 17, 2024 | large-billed crow | G2d-0 | A/large-billed crow/Hokkaido/0112F077/2024 | EPI_ISL_19776381 | Kagoshima University |
| 91 | Kagoshima | December 17, 2024 | white-naped crane | G2d-0 | A/white-naped crane/Kagoshima/KU-24-66/2024 | EPI_ISL_19767062 | Kagoshima University |
| 92 | Hokkaido | December 20, 2024 | large-billed crow | G2d-0 | A/large-billed crow/Hokkaido/0112F078/2024 | EPI_ISL_19772333 | Kagoshima University |
| 93 | Hokkaido | December 20, 2024 | large-billed crow | G2d-0 | A/large-billed crow/Hokkaido/0112N170/2024 | EPI_ISL_19772332 | Kagoshima University |
| 94 | Hokkaido | December 24, 2024 | large-billed crow | G2d-0 | A/large-billed crow/Hokkaido/0112F079/2024 | EPI_ISL_19835913 | Hokkaido University |
| 95 | Kochi | December 25, 2024 | Eastern buzzard | G2d-0 | A/eastern buzzard/Kochi/3912T001T/2025 | EPI_ISL_20111854 | National Institute of Animal Health |
| 96 | Iwate | December 26, 2024 | Eastern buzzard | G2d-9 | A/eastern buzzard/Iwate/0312D004/2024 | EPI_ISL_19835916 | Hokkaido University |
| 97 | Hokkaido | December 26, 2024 | carrion crow | G2d-0 | A/carrion_crow/Hokkaido/0112Q115/2024 | EPI_ISL_20095325 | Tottori University |
| 98 | Kagoshima | December 27, 2024 | northern goshawk | G2d-0 | A/northern_goshawk/Kagoshima/KU-24-73/2024 | EPI_ISL_19767063 | Kagoshima University |
| 99 | Aichi | January 2, 2025 | large-billed crow | G2d-0 | A/large-billed crow/Aichi/TU17-47/2025 | EPI_ISL_19741665 | Tottori University |
| 100 | Hokkaido | January 6, 2025 | large-billed crow | G2d-0 | A/large-billed_crow/Hokkaido/0101F081/2025 | EPI_ISL_20095326 | Tottori University |
| 101 | Saitama | January 6, 2025 | mandarin duck | G2d-0 | A/mandarin duck/Saitama/1101A005/2025 | EPI_ISL_19835910 | Hokkaido University |
| 102 | Kagoshima | January 10, 2025 | hooded crane | G2c-17 | A/hooded crane/Kagoshima/KU-24-82/2024 | EPI_ISL_19767064 | Kagoshima University |
| 103 | Hokkaido | January 14, 2025 | carrion crow | G2d-0 | A/carrion_crow/Hokkaido/0101Q116/2025 | EPI_ISL_20095327 | Tottori University |
| 104 | Kagoshima | January 16, 2025 | hooded crane | G2c-17 | A/hooded crane/Kagoshima/KU-24-84/2024 | EPI_ISL_19769504 | Kagoshima University |
| 105 | Kagoshima | January 18, 2025 | Eurasian wigeon | G2c-17 | A/Eurasian_wigeon/Kagoshima/KU-24-4/2024 | EPI_ISL_19645341 | Kagoshima University |
| 106 | Kagoshima | January 20, 2025 | hooded crane | G2c-17 | A/hooded crane/Kagoshima/KU-24-87/2024 | EPI_ISL_19769505 | Kagoshima University |
| 107 | Kagoshima | January 21, 2025 | hooded crane | G2c-17 | A/hooded crane/Kagoshima/KU-24-88/2024 | EPI_ISL_19850327 | Kagoshima University |
| 108 | Kagoshima | February 3, 2025 | hooded crane | G2c-17 | A/hooded crane/Kagoshima/KU-24-93/2024 | EPI_ISL_19850334 | Kagoshima University |
| 109 | Hokkaido | February 14, 2025 | large-billed crow | G2d-0 | A/large-billed crow/Hokkaido/B172/2025 | EPI_ISL_19750619 | Hokkaido University |
| 110 | Kagoshima | February 16, 2025 | hooded crane | G2c-17 | A/hooded crane/Kagoshima/KU-24-103/2024 | EPI_ISL_19850329 | Kagoshima University |
| 111 | Kagoshima | February 16, 2025 | hooded crane | G2c-17 | A/hooded crane/Kagoshima/KU-24-104/2024 | EPI_ISL_19850330 | Kagoshima University |
| 112 | Kagoshima | February 17, 2025 | white-naped crane | G2c-17 | A/white-naped crane/Kagoshima/KU-24-105/2024 | EPI_ISL_19850331 | Kagoshima University |
| 113 | Hokkaido | February 20, 2025 | large-billed crow | G2d-0 | A/large-billed crow/Hokkaido/B175/2025 | EPI_ISL_19906326 | Hokkaido University |
| 114 | Hokkaido | February 25, 2025 | carrion crow | G2d-0 | A/carrion crow/Hokkaido/0102Q118T/2025 | EPI_ISL_20111873 | National Institute of Animal Health |
| 115 | Hokkaido | February 28, 2025 | large-billed crow | G2d-0 | A/large-billed crow/Hokkaido/B176/2025 | EPI_ISL_19888043 | Hokkaido University |
| 116 | Hokkaido | February 28, 2025 | large-billed crow | G2d-0 | A/large-billed crow/Hokkaido/0102B135C/2025 | EPI_ISL_20111859 | National Institute of Animal Health |
| 117 | Hokkaido | March 3, 2025 | large-billed crow | G2d-0 | A/large-billed crow/Hokkaido/0103B136T/2025 | EPI_ISL_20111934 | National Institute of Animal Health |
| 118 | Hokkaido | March 10, 2025 | large-billed crow | G2d-0 | A/large-billed crow/Hokkaido/B188/2025 | EPI_ISL_19906342 | Hokkaido University |
| 119 | Hokkaido | March 11, 2025 | large-billed crow | G2d-0 | A/large-billed crow/Hokkaido/0103B137/2025 | EPI_ISL_19835914 | Hokkaido University |
| 120 | Hokkaido | March 11, 2025 | large-billed crow | G2d-0 | A/large-billed_crow/Hokkaido/B190/2025 | EPI_ISL_20127437 | Hokkaido University |
| 121 | Iwate | March 11, 2025 | large-billed crow | G2d-0 | A/large-billed crow/Iwate/0303F002/2025 | EPI_ISL_19835911 | Hokkaido University |
| 122 | Iwate | March 12, 2025 | large-billed crow | G2d-0 | A/large-billed crow/Iwate/0303F001/2025 | EPI_ISL_19835912 | Hokkaido University |
| 123 | Fukushima | March 12, 2025 | Eastern buzzard | G2d-9 | A/eastern buzzard/Fukushima/0703A001/2025 | EPI_ISL_19835917 | Hokkaido University |
| 124 | Hokkaido | March 12, 2025 | large-billed crow | G2d-0 | A/large-billed crow/Hokkaido/0103F083T/2025 | EPI_ISL_20111937 | National Institute of Animal Health |
| 125 | Hokkaido | March 14, 2025 | large-billed crow | G2d-0 | A/large-billed crow/Hokkaido/B191/2025 | EPI_ISL_19906344 | Hokkaido University |
| 126 | Hokkaido | March 14, 2025 | whooper swan | G2d-0 | A/whooper swan/Hokkaido/0103Q122T/2025 | EPI_ISL_20111939 | National Institute of Animal Health |
| 127 | Hokkaido | March 15, 2025 | large-billed crow | G2d-0 | A/large-billed crow/Hokkaido/B192/2025 | EPI_ISL_19888044 | Hokkaido University |
| 128 | Iwate | March 18, 2025 | large-billed crow | G2d-0 | A/large-billed crow/Iwate/0303I002T/2025 | EPI_ISL_20111941 | National Institute of Animal Health |
| 129 | Iwate | March 18, 2025 | large-billed crow | G2d-0 | A/large-billed crow/Iwate/0303I003T/2025 | EPI_ISL_20111943 | National Institute of Animal Health |
| 130 | Hokkaido | March 20, 2025 | large-billed crow | G2d-0 | A/large-billed crow/Hokkaido/B193/2025 | EPI_ISL_19906347 | Hokkaido University |
| 131 | Hokkaido | March 21, 2025 | large-billed crow | G2d-0 | A/large-billed crow/Hokkaido/B196/2025 | EPI_ISL_19906348 | Hokkaido University |
| 132 | Hokkaido | March 21, 2025 | large-billed crow | G2d-0 | A/large-billed crow/Hokkaido/B198/2025 | EPI_ISL_19906349 | Hokkaido University |
| 133 | Hokkaido | March 22, 2025 | crested auklet | G2d-0 | A/crested auklet/Hokkaido/P001/2025 | EPI_ISL_19855235 | Hokkaido University |
| 134 | Hokkaido | March 22, 2025 | crested auklet | G2d-0 | A/crested auklet/Hokkaido/P002/2025 | EPI_ISL_19855236 | Hokkaido University |
| 135 | Hokkaido | March 22, 2025 | crested auklet | G2d-0 | A/crested auklet/Hokkaido/P003/2025 | EPI_ISL_19855237 | Hokkaido University |
| 136 | Hokkaido | March 22, 2025 | ancient murrelet | G2d-0 | A/ancient murrelet/Hokkaido/P004/2025 | EPI_ISL_19855238 | Hokkaido University |
| 137 | Iwate | March 23, 2025 | large-billed crow | G2d-0 | A/large-billed crow/Iwate/0303G003/2025 | EPI_ISL_19835918 | Hokkaido University |
| 138 | Iwate | March 23, 2025 | large-billed crow | G2d-0 | A/large-billed crow/Hokkaido/B199/2025 | EPI_ISL_19906350 | Hokkaido University |
| 139 | Fukushima | March 24, 2025 | large-billed crow | G2d-0 | A/large-billed crow/Fukushima/0703B002/2025 | EPI_ISL_19835915 | Hokkaido University |
| 140 | Hokkaido | March 24, 2025 | large-billed crow | G2d-0 | A/large-billed crow/Hokkaido/0103B139C/2025 | EPI_ISL_20111950 | National Institute of Animal Health |
| 141 | Fukushima | March 25, 2025 | large-billed crow | G2d-0 | A/large-billed crow/Fukushima/0703B005C/2025 | EPI_ISL_20111945 | National Institute of Animal Health |
| 142 | Iwate | March 25, 2025 | large-billed crow | G2d-0 | A/large-billed crow/Iwate/0303F005T/2025 | EPI_ISL_20111946 | National Institute of Animal Health |
| 143 | Iwate | March 25, 2025 | large-billed crow | G2d-0 | A/large-billed crow/Iwate/0303G004T/2025 | EPI_ISL_20111948 | National Institute of Animal Health |
| 144 | Hokkaido | March 25, 2025 | slaty-backed gull | G2d-0 | A/slaty-backed gull/Hokkaido/0103P128T/2025 | EPI_ISL_20111959 | National Institute of Animal Health |
| 145 | Hokkaido | March 25, 2025 | slaty-backed gull | G2d-0 | A/slaty-backed gull/Hokkaido/0103P129T/2025 | EPI_ISL_20111960 | National Institute of Animal Health |
| 146 | Hokkaido | March 25, 2025 | large-billed crow | G2d-0 | A/large-billed crow/Hokkaido/0103F084T/2025 | EPI_ISL_20111962 | National Institute of Animal Health |
| 147 | Hokkaido | March 26, 2025 | large-billed crow | G2d-0 | A/large-billed crow/Hokkaido/B201/2025 | EPI_ISL_19888057 | Hokkaido University |
| 148 | Hokkaido | March 29, 2025 | crow | G2d-0 | A/crow/Hokkaido/B205/2025 | EPI_ISL_20127467 | Hokkaido University |
| 149 | Hokkaido | March 30, 2025 | carrion crow | G2d-0 | A/carrion crow/Hokkaido/0103Q127T/2025 | EPI_ISL_20111951 | National Institute of Animal Health |
| 150 | Hokkaido | March 30, 2025 | carrion crow | G2d-0 | A/carrion crow/Hokkaido/0104Q128T/2025 | EPI_ISL_20111953 | National Institute of Animal Health |
| 151 | Hokkaido | March 30, 2025 | large-billed crow | G2d-0 | A/large-billed_crow/Hokkaido/B207/2025 | EPI_ISL_20127492 | Hokkaido University |
| 152 | Hokkaido | March 31, 2025 | large-billed crow | G2d-0 | A/large-billed_crow/Hokkaido/B211/2025 | EPI_ISL_20127549 | Hokkaido University |
| 153 | Iwate | March 31, 2025 | black kite | G2d-0 | A/black kite/Iwate/0303H001T/2025 | EPI_ISL_20112948 | National Institute of Animal Health |
| 154 | Hokkaido | April 2, 2025 | carrion crow | G2d-0 | A/carrion crow/Hokkaido/0104Q129T/2025 | EPI_ISL_20111955 | National Institute of Animal Health |
| 155 | Hokkaido | April 4, 2025 | slaty-backed gull | G2d-0 | A/slaty-backed gull/Hokkaido/0104Q130T/2025 | EPI_ISL_20111957 | National Institute of Animal Health |
| 156 | Hokkaido | April 7, 2025 | spectacled guillemot | G2d-0 | A/spectacled guillemot/Hokkaido/20250331001T/2025 | EPI_ISL_20112950 | National Institute of Animal Health |
| 157 | Hokkaido | April 8, 2025 | large-billed crow | G2d-0 | A/large-billed crow/Hokkaido/0104P134/2025 | EPI_ISL_19908842 | Hokkaido University |
| 158 | Hokkaido | April 8, 2025 | carrion crow | G2d-0 | A/carrion crow/Hokkaido/B220/2025 | EPI_ISL_20127556 | Hokkaido University |
| 159 | Hokkaido | April 12, 2025 | common murre | G2d-0 | A/common murre/Hokkaido/20250410002T/2025 | EPI_ISL_20112952 | National Institute of Animal Health |
| 160 | Hokkaido | April 12, 2025 | common murre | G2d-0 | A/common murre/Hokkaido/20250410003T/2025 | EPI_ISL_20112953 | National Institute of Animal Health |
| 161 | Hokkaido | April 14, 2025 | large-billed crow | G2d-0 | A/large-billed crow/Hokkaido/B225/2025 | EPI_ISL_19906351 | Hokkaido University |
| 162 | Hokkaido | April 15, 2025 | black-tailed gull | G2d-0 | A/black-tailed gull/Hokkaido/0104P135T/2025 | EPI_ISL_20112955 | National Institute of Animal Health |
| 163 | Hokkaido | April 16, 2025 | ancient murrelet | G2d-0 | A/ancient murrelet/Hokkaido/20250415002T/2025 | EPI_ISL_20112956 | National Institute of Animal Health |
| 164 | Hokkaido | April 16, 2025 | spectacled guillemot | G2d-0 | A/spectacled guillemot/Hokkaido/20250415003T/2025 | EPI_ISL_20112958 | National Institute of Animal Health |
| 165 | Iwate | April 16, 2025 | large-billed crow | G2d-0 | A/large-billed crow/Iwate/0304I003/2025 | EPI_ISL_19908841 | Hokkaido University |
| 166 | Hokkaido | April 16, 2025 | carrion crow | G2d-0 | A/carrion crow/Hokkaido/0104Q140/2025 | EPI_ISL_19908843 | Hokkaido University |
| 167 | Hokkaido | April 16, 2025 | crested auklet | G2d-0 | A/crested auklet/Hokkaido/0104Q142/2025 | EPI_ISL_19908844 | Hokkaido University |
| 168 | Hokkaido | April 17, 2025 | large-billed crow | G2d-0 | A/large-billed crow/Hokkaido/B226/2025 | EPI_ISL_19888058 | Hokkaido University |
| 169 | Hokkaido | April 20, 2025 | large-billed crow | G2d-0 | A/large-billed crow/Hokkaido/B227/2025 | EPI_ISL_19888059 | Hokkaido University |
| 170 | Hokkaido | April 20, 2025 | large-billed crow | G2d-0 | A/large-billed_crow/Hokkaido/B228/2025 | EPI_ISL_20127601 | Hokkaido University |
| 171 | Hokkaido | April 22, 2025 | large-billed crow | G2d-0 | A/large-billed crow/Hokkaido/B229/2025 | EPI_ISL_19888060 | Hokkaido University |
| 172 | Hokkaido | April 28, 2025 | large-billed crow | G2d-0 | A/large-billed crow/Hokkaido/0104E106/2025 | EPI_ISL_19908915 | Hokkaido University |
| 173 | Hokkaido | May 1, 2025 | Gavia spp. | G2d-0 | A/*Gavia* spp./Hokkaido/20250501004/2025 | EPI_ISL_19908916 | Hokkaido University |
| 174 | Hokkaido | May 2, 2025 | red-necked grebe | G2d-0 | A/red-necked grebe/Hokkaido/20250501007C/2025 | EPI_ISL_20112960 | National Institute of Animal Health |
| 175 | Hokkaido | May 2, 2025 | rhinoceros auklet | G2d-0 | A/rhinoceros auklet/Hokkaido/20250501010T/2025 | EPI_ISL_20112961 | National Institute of Animal Health |
| 176 | Hokkaido | June 17, 2025 | white-tailed eagle | G2d-0 | A/white-tailed_eagle/Hokkaido/20250617B1/2025 | EPI_ISL_20086275 | Hokkaido University |

**Table S3. Representative isolates with gene segments closely related to those detected in Japan during the 2024–2025 season**

| Gene Segment | Cluster ID | Representative strain | Subtype | Country | Collection date | GISAID accession no. |
| --- | --- | --- | --- | --- | --- | --- |
| PB2 | AIV-PB2-VII | A/bean goose/Korea/KNU-28/2023 | H6N8 | South Korea | 4 April, 2023 | EPI_ISL_18698943 |
|  | AIV-PB2-VIII | A/spot-billed duck/South Korea/KNU2022-73/2022 | H4N6 | South Korea | 31 October, 2022 | EPI_ISL_19109827 |
|  | AIV-PB2-IX | A/*Cygnus columbianus*/Hubei/117/2021 | H5N1 | China | 20 December, 2021 | EPI_ISL_11633613 |
| PB1 | AIV-PB1-VI | A/spot billed duck/Korea/KNU-30/2022 | H1N1 | South Korea | 11 October, 2022 | EPI_ISL_18698826 |
|  | AIV-PB1-VII | A/mallard/Novosibirsk region/3524k/2020 | H4N6 | Russia | 29 August, 2020 | EPI_ISL_1241000 |
|  | AIV-PB1-VIII | A/wild bird/South Korea/Q23-393/2023 | H9N2 | South Korea | 24 October, 2023 | EPI_ISL_18900625 |
| PA | AIV-PA-IX | A/common teal/Guangdong/352/2023 | H4N6 | China | 1 November, 2023 | EPI_ISL_19859450 |
|  | AIV-PA-X | A/ruddy shelduck/Qinghai/JXG-721-19/2023 | H10N5 | China | 21 July, 2023 | EPI_ISL_19214962 |
|  | AIV-PA-XI | A/pintail/South Korea/KNU20-45/2020 | H5N2 | South Korea | 18 March, 2020 | EPI_ISL_19109844 |
|  | AIV-PA-XII | A/swan/Shanxi/2143/2023 | H12N2 | China | 21 November, 2023 | EPI_ISL_19226689 |
| NP | AIV-NP-VI | A/gadwall/Novosibirsk region/3407k/2020 | H4N6 | Russia | 29 August, 2020 | EPI_ISL_1184520 |
|  | AIV-NP-VII | A/bean goose/Korea/KNU-09/2023 | H6N2 | South Korea | 6 March, 2023 | EPI_ISL_18698814 |
|  | AIV-NP-VIII | A/mandarin duck/Korea/24WS005-2/2024 | H5N1 | South Korea | 14 October, 2024 | EPI_ISL_19528860 |
|  | AIV-NP-IX | A/common teal/Guangdong/352/2023 | H4N6 | China | 1 November, 2023 | EPI_ISL_19859450 |
|  | AIV-NP-X | A/wild bird/South Korea/E23-931/2023 | H6N8 | South Korea | 5 December, 2023 | EPI_ISL_18900618 |
|  | AIV-NP-XI | A/northern pintail/Russia Primorje/96T/2020 | H3N8 | Russia | 3 October, 2020 | EPI_ISL_1241003 |
| NS | AIV-NS-III | A/common teal/Guangdong/60/2023 | H4N6 | China | 1 November, 2023 | EPI_ISL_19859452 |
|  | AIV-NS-IV | A/duck/Bangladesh/19D2067/2023 | H9N9 | Bangladesh | 18 January, 2023 | EPI_ISL_18983962 |
|  | AIV-NS-V | A/wild bird/Guangdong/ZJ476/2022 | H4N6 | China | 20 November, 2022 | EPI_ISL_19859439 |

| Isolate | Genotype | Subtype | Season | CLD_50_ ^*^ (log_10_EID_50_) | 10^6^ EID_50_ inoculation group | | | | | | |
| --- | --- | --- | --- | --- | --- | --- | --- | --- | --- | --- | --- |
|  |  |  |  |  | MDT ^†^ | | Mean maximum viral titer (log_10_EID_50_/mL) ^¶^ | | AUC of entire observation period # | | |
|  |  |  |  |  | hpi ^‡^ | dpi ^§^ | Trachea | Cloaca | Trachea | Cloaca | Trachea and cloaca |
| A/chicken/Kagawa/22B2T/2022 | G2c-6 | H5N1 | 2022–23 | 5.50 | 73.50 | 3.06 | 4.29 | 2.32 | 18092 | 226 | 18319 |
|  |  |  |  |  | (72.00–78.00) | (3.00–3.25) | (3.53–4.87) | (2.15–2.41) | (3858–36903) | (85–565) | (4423–37014) |
| A/chicken/Aichi/22A3T/2022 | G2c-8 | H5N1 | 2022–23 | 4.30 | 63.60 | 2.65 | 6.27 | 3.95 | 2066596 | 14912 | 2081509 |
|  |  |  |  |  | (48.00–72.00) | (2.00–3.00) | (5.53–6.87) | (2.53–4.87) | (170324–4538605) | (509–36714) | (17625–4558084) |
| A/chicken/Hiroshima/22A6T/2022 | G2c-9 | H5N1 | 2022–23 | 4.70 | 148.80 | 6.20 | 4.89 | 3.12 | 181978 | 4061 | 186039 |
|  |  |  |  |  | (144.00–168.00) | (6.00–7.00) | (4.32–5.53) | (1.70–4.20) | (23056–547400) | (49–8653) | (30977–556053) |
| A/chicken/Kagoshima/23B1T/2024 ^**^ | G2c-12 | H5N6 | 2023–24 | 4.60 | 54.00 | 2.25 | 5.37 | 4.68 | 211945 | 43784 | 255729 |
|  |  |  |  |  | (48.00–72.00) | (2.00–3.00) | (4.53–5.87) | (4.20–5.10) | (25791–373155) | (7905–96599) | (85761–469754) |

**Table S4. Virological characteristics of H5N1 high pathogenicity avian influenza (HPAI) viruses during the 2022–2023 and 2023-2024 seasons**

*CLD_50_, 50% chicken lethal dose

EID_50_, 50% egg infectious dose

†MDT, mean death time

‡hpi, hours post-inoculation; values in parentheses, range of hpi

§dpi, days post-inoculation; values in parentheses, range of dpi

¶Numbers in parentheses, range of maximum viral titers

\# AUC, area under the curve; values in parentheses, range of AUC

**CLD_50_, MDT, and maximum viral titer of this virus were clarified in our previous study (Nishiura et al., 2024). The AUC of this virus was calculated in the present study.

**Table S5. Amino acid mutations associated with pathogenicity of high pathogenicity avian influenza (HPAI) viruses**

| Protein | Amino acid mutation* | G2d-0: Chiba/24A2T | G2d-5: Niigata/24B3T | G2d-6: Gifu/24A5T | G2c-6: Kagawa/22B2T | G2c-8: Aichi/22A3T | G2c-9: Hiroshima/22A6T | G2c-12: Kagoshima/23B1T | G2c-13: Chiba/24B7T | G2c-14: Ehime/24A3T | Phenotype |
| --- | --- | --- | --- | --- | --- | --- | --- | --- | --- | --- | --- |
| HA | S133A | + | + | + | + | + | + | + | + | + | Increased binding to α2-6 |
|  | S154N | + | + | + | - | - | - | - | - | - | Increased binding to α2-6 |
|  | N154D | - | - | - | + | - | + | + | + | + | Decreased virulence in mice |
|  | T156A | + | + | + | + | + | + | + | + | + | Increased binding to α2-6 |
|  | V182N | + | + | + | + | + | + | + | + | + | Increased binding to α2-6; decreased binding to α2-3 |
|  | K394E | + | + | + | - | - | - | - | + | + | Increased pH of fusion; decreased HA stability; decreased virulence in mice |
|  | S107R and T108I | + | + | + | + | + | + | + | + | + | Increased virulence in chickens and mice; increased pH of fusion |
|  | K218Q and S223R | + | + | + | + | + | + | + | + | + | Increased binding to α2-6 and α2-3 |
| PB2 | I292V | - | - | + | - | - | - | - | + | - | Increased polymerase activity in mammalian cell line; increased virulence in mice |
|  | K389R | + | + | + | + | + | + | + | + | + | Increased polymerase activity and replication in mammalian cell line |
|  | K482R | + | - | - | - | - | - | - | - | - | Increased polymerase activity in mammalian cell line |
|  | V598T | + | + | + | + | + | + | + | + | + | Increased polymerase activity and replication in mammalian cells; increased virulence in mice |
|  | K627E | + | + | + | + | + | + | + | + | + | Increased virulence in chickens |
|  | S715N | + | + | + | + | + | + | + | + | + | Decreased virulence in mice |
|  | L89V and G309D | + | + | + | + | + | + | + | + | + | Increased polymerase activity in mammalian cell line; increased virulence in mice |
|  | L89V, G309D, T339K, R477G, I495V, K627E, and A676T | + | + | + | + | + | + | + | + | - | Increased polymerase activity in mammalian cell line; increased virulence in mice |
| PB1 | D3V | + | + | + | + | + | + | + | + | + | Increased polymerase activity and viral replication in avian and mammalian cell lines |
|  | D622G | + | + | + | + | + | + | + | + | + | Increased polymerase activity and virulence in mice |
| PB1-F2 | N66S | + | + | - | + | + | + | + | - | + | Enhanced replication; virulence and antiviral response in mice |
| PA | S37A | + | + | + | + | + | + | + | + | + | Increased polymerase activity in mammalian cell line |
|  | P190S | + | + | + | + | + | + | + | + | + | Decreased virulence in mice |
|  | N383D | + | + | + | + | + | + | + | + | - | Increased polymerase activity in mammalian and avian cell lines |
|  | Q400P | + | - | - | - | + | - | - | - | + | Decreased virulence in mice |
|  | N409S | + | + | + | + | + | + | + | + | + | Increased polymerase activity and replication in mammalian cell line |
| NP | M105V | + | - | + | - | + | - | - | + | + | Increased virulence in chickens |
|  | A184K | + | + | + | + | + | + | + | + | + | Increased replication in avian cells and virulence in chickens; enhanced IFN response |
|  | N319K | + | - | - | - | - | - | - | - | - | Increased polymerase activity and replication in mammalian cell line |
| NS1 | P42S | + | - | + | - | + | + | + | + | + | Increased virulence and decreased antiviral response in mice |
|  | I106M | + | + | + | + | + | + | + | + | + | Increased viral replication in mammalian cells virulence in mice |
|  | C138F | + | + | + | + | + | + | + | + | + | Increased replication in mammalian cells; decreased interferon response |
|  | V149A | + | + | + | + | + | + | + | + | + | Increased virulence and decreased interferon response in chickens |
|  | L103F and I106M | + | - | + | - | + | + | + | + | + | Increased virulence in mice |
|  | K55E, K66E, and C138F | + | - | + | - | + | + | + | + | + | Enhanced replication in mammalian cells; decrease in IF response |
|  | 222–230 deletion | - | - | - | - | + | - | - | - | + | Increased replication in mammalian and avian cell lines |
|  | ^227^ESEV^230^ | + | - | + | + | - | + | + | + | - | Decreased viral replication in mammalian and avian cell lines |
| M1 | N30D | + | + | + | + | + | + | + | + | + | Increased virulence in mice |
|  | I43M | + | + | + | + | + | + | + | + | + | Increased virulence in mice, chickens, and ducks |
|  | T215A | + | + | + | + | + | + | + | + | + | Increased virulence in mice |

*Pathogenicity markers of AIVs reported in previous study (Suttie et al, 2019).
